# Impact of volatile organic compounds on chromium containing atmospheric particulate: insights from molecular dynamics simulations

**DOI:** 10.1038/s41598-020-74522-x

**Published:** 2020-10-15

**Authors:** Dhawal Shah, Mirat Karibayev, Enoch Kwasi Adotey, Mehdi Amouei Torkmahalleh

**Affiliations:** Department of Chemical and Materials Engineering, School of Engineering and Digital Sciences, Nazarbayev University, 010000 Nur-Sultan, Kazakhstan

**Keywords:** Climate sciences, Environmental sciences

## Abstract

The effect of volatile organic compounds (VOCs) on chromium-containing atmospheric particles remains obscured because of difficulties in experimental measurements. Moreover, several ambiguities exist in the literature related to accurate measurements of atmospheric chromium concentration to evaluate its toxicity. We investigated the interaction energies and diffusivity for several VOCs in chromium (III)-containing atmospheric particles using classical molecular dynamics simulations. We analyzed xylene, toluene, ascorbic acid, carbon tetrachloride, styrene, methyl ethyl ketone, naphthalene, and anthracene in Cr(III) solutions, with and without air, to compare their effects on solution chemistry. The interaction energy between Cr(III) and water changed from 48 to 180% for different VOCs, with the highest change with anthracene and the lowest change with naphthalene. The results revealed no direct interactions between Cr(III) particles and the analyzed volatile organic compounds, except ascorbic acid. Interactions of Cr(III) and ascorbic acid differ significantly between the solution phase and the particulate phase. The diffusion of Cr(III) and all the VOCs also were observed in a similar order of magnitude (~ 10^−5^ cm^2^/s). The results can further assist in exploring the variation in chromium chemistry and reaction rates in the atmospheric particles in the presence of VOCs.

## Introduction

Chromium exists naturally in the atmosphere through activities such as volcanic eruptions, erosion from soils, and rocks^1–3^ . Seigneur and Constantinou^4^ reported that approximately 65% of atmospheric chromium has anthropogenic origins. Anthropogenic activities, such as electroplating, leather tanning industries, metal polishing, refractory production, and fuel combustion, contribute to the atmospheric chromium^5^. Wide-spread use of chromium and its associated ores has led to environmental pollutions, including the formation of airborne chromium. For example, soluble Cr(VI) concentrations in the ambient air of Paterson (0.44 ± 0.35 ng/m^3^) and Chester (0.40 ± 0.53 ng/m^3^), USA, which are an industrial city and a background site, respectively, were reported by Fan et al.^6^. In Radom, Poland, Molik et al.^7^ reported total Cr and Cr(VI) concentrations to be 25 and 6 ng/m^3^, respectively, with a Cr(VI)/total Cr ratio as 36%. The ERG^8^ also conducted Cr(VI) measurements over 22 sites and reported that the concentration of soluble Cr(VI) was in the range of 0.001 to 2.97 ng/m^3^, with an average Cr(VI) concentration of 0.044 ng/m^3^. Field sampling conducted by Amouei Torkmahalleh et al.^9^ using an improved chromium sampler in New Jersey, USA, also reported the average ambient Cr(VI) concentrations of 0.03 ± 0.01 ng m^−3^ and 0.02 ± 0.01 ng m^−3^, for summer and winter periods respectively. Canepari et al.^10^ also carried out measurements at the peri-urban site near Rome and reported the total Cr concentration in PM_10_ in the range of 2–5 ng/m^3^, having a 5–13% soluble fractions. Additionally, in the industrial area of Northern Italy, they found the total Cr concentration ranging from 6 to 11 ng/m^3^, the soluble fraction of about 11–28%, and a Cr(VI)/Cr(III) ratio in the range of 0.5–2.5. The soluble and total Cr(VI) concentrations in ambient PM_10_ collected with mixed Cr emission sources in New Jersey^11^ recorded the mean concentrations of 1.05–1.41 ng/m^3^ (winter) and 0.99–1.56 ng/m^3^ (summer) for total Cr(VI); 0.11–0.19 ng/m^3^ (winter) and 0.18–0.37 ng/m^3^ (summer) for soluble Cr(VI). Likewise, in a suburb area they reported the mean concentrations of 1.07 ng/m^3^ (winter) and 0.99 ng/m^3^ (summer) for total Cr(VI); 0.03 ng/m^3^ (winter) and 0.12 ng/m^3^ (summer) for soluble Cr(VI)^11^.

In the atmosphere, chromium exists in two stable states; trivalent chromium, Cr(III), and hexavalent chromium, Cr(VI)^5^. Cr(III) is primarily essential for living organisms by controlling the metabolisms of lipid and glucose^5^. Cr(VI), on the other hand, is a known carcinogen and leads to adverse health effects such as perforation of the nasal septum, asthmatic conditions, bronchitis, pneumonitis, and lung cancer^5^. Slightly soluble and highly insoluble Cr(VI) particles such as the chromates of zinc, lead, strontium, barium, and sintered calcium induce a tumor response on animals^12^. Insoluble forms of the Cr(VI), when deposited in the lung, are known to impose health effects, chronically^13^. Soluble Cr (VI) is known to enter the bloodstream and is later excreted or converted to Cr(III) in the bloodstream^13^. Recent works are being tailored to investigate the reactivity of Cr in the atmosphere to better understand its speciation^14,15^.

Additionally, the valence state of Cr in the atmosphere also depends on the solution equilibrium between Cr(III) and Cr(VI). The interconversion between these two species occurs in solution as well as via solid–gas reactions^1,9^. Moreover, the interconversion between Cr(VI) and Cr(III) can also occur in atmosphere in the presence of heavy metals like vanadium and iron, and gases including SO_2_ and NO_2_^1,16,17^. Due to complex nature on the concept of atmospheric chemistry, reactions between chromium and atmospheric particles are further investigated. For example, Schroeder and Lee^18^ reported oxidation of Cr(III) to Cr(VI) in the presence of Mn(III) (see Eq. 1). Iron (II), according to Pettine and Tonnina^19^, was identified to favor the reduction of Cr(VI) to Cr(III) (Eq. 2). Various reduced sulfur such as S, S^2−^, H_2_S, HSO_3_^−^ and S_2_O_3_, were also known to reduce Cr(VI) to Cr(III) (Eq. 3). Reaction with H_2_O_2_ is also known to reduce Cr(VI) to Cr(III) at pH < 7.5^20^.

1 $${\text{2Cr}}\left( {{\text{III}}} \right) + {\text{3Mn}}\left( {{\text{IV}}} \right) \rightleftharpoons {\text{2Cr}}\left( {{\text{VI}}} \right) + {\text{3Mn}}\left( {{\text{II}}} \right)$$

2 $${\text{Cr}}\left( {{\text{VI}}} \right) + {\text{3Fe}}\left( {{\text{II}}} \right) \rightleftharpoons {\text{Cr}}\left( {{\text{III}}} \right) + {\text{3Fe}}\left( {{\text{III}}} \right)$$

3 $${\text{2HCrO}}_{{4\;({\text{aq}})}}^{ - } + {\text{ 4HSO}}_{3}^{ - } + {\text{ 6H}}^{ + } \rightleftharpoons {\text{2Cr}}^{{{3} + }} + {\text{ 2SO}}_{{4}}^{ - } + {\text{ S}}_{{2}} {\text{O}}_{{6}}^{{{2} - }} + {\text{6H}}_{{2}} {\text{O}}$$

While the prior studies conducted on Cr speciation considered groundwater conditions and were centralized on the presence of reductants or oxidants available in the aqueous medium, our understanding of chromium reactions with volatile organic compounds in particulate matter under atmospheric conditions is limited. Recently, Amouei Torkmahalleh et al.^21^ developed a computer model by utilizing the earlier concept of Seigneur and Constantinou^4^. They implemented field data collected in New Jersey, US, to understand the chemistry and speciation of soluble, and insoluble chromium at pH ~ 9. Simulation results showed that Cr(VI) was dominant as CrO_4_^2−^ in the soluble form and (NH_4_)_2_CrO_4_, CaCrO_4_, BaCrO_4_, and PbCrO_4_ were in the precipitated form. The reduction rate of Cr(VI) to Cr(III) was observed to be higher than the oxidation rate of Cr(III) to Cr(VI). The use of a basic pH solution was found to retard the conversion of Cr(VI) in the presence of Fe(II) and As(III) and though it facilitated the precipitation of Cr(III)^21^. Although dissolved gases such as O_3_, O_2_, HO_2_, and HO_3_ were employed in their model, possible reaction of VOCs were not included to understand Cr chemistry under typical atmospheric conditions.

A model of Cr speciation in deliquesced particles (pH ~ 4) was developed by Konakbayeva et al. to provide more insight into the soluble and insoluble forms of Cr atmospheric particles^14^. Simulation results indicated that the insoluble Cr(III) compound, Cr_2_(SO_4_)_3(s)_, was the dominant form of Cr(III) at pH ~ 4, and the overall conversion direction was identified as from Cr(VI) to Cr(III).

Chromium species are known to react with VOCs, ozone, and with other chemical species under atmospheric conditions. It is thus imperative to study the reactions of dissolved VOCs with chromium. Volatile organic compounds are common atmospheric pollutants primarily emitted from petrochemical, chemical, and other industries. There are various photochemical reactions that take place in the presence of VOCs and lead to the formation of different environmental hazards^15,22,23^. Various VOCs have been reported under atmospheric conditions with concentrations in the order of mg/${\text{m}}_{{{\text{air}}}}^{3}$. Nonpolar VOCs can be classified as aliphatic compounds (e.g., methane, hexane, and heptane), halogenated hydrocarbons (e.g., chloroform, carbon tetrachloride and chlorobenzenes), and aromatic compounds. Under atmospheric conditions, about 60–90% of these aromatic compounds include benzene, toluene, xylenes, ethylbenzene, and 1,2,4-trimethylbenzene. Polar VOCs include acetone, ethyl acetate, methyl isobutyl ketone, butyl acetate, ethanol, propanol, etc.,^24–26^. The photolysis and the initial reactions of the VOCs with OH radicals and NO_3_ radicals leading to the formation of alkyl or substituted alkyl (RO) radicals have been given much attention^23^. However, the intermolecular interactions of these VOCs with airborne particulate matter containing species such as Cr(III), have barely been investigated.

Shah et al.^27^ initially studied the molecular dynamic simulation of atmospheric chromium (III) compound in the presence of additives such as ozone, formaldehyde, and benzene. The simulation studied different scenarios with Cr(III)-containing particles as well as the effect of air around atmospheric particles to deduce that no direct interaction between Cr(III) and the additives existed. However, the presence of these additives altered Cr(III)/water interactions. Although the diffusion of Cr(III) and the additives were observed to be fast, they concluded that diffusion was not a controlling factor to influence the chemistry in atmospheric particles. When particles undergo deliquescence, VOCs react with Cr(VI) in the solution, thereby reducing it to Cr(III), and subsequent interactions of Cr(III) with VOCs can impact the availability of the VOCs molecules in the medium. There is, therefore, the possibility that reaction rate could be altered due to these interactions. Understanding the chemistry of Cr(III) with other VOCs is necessary, as well as, studying the physical properties of chromium (III) in atmospheric particles in the presence of dissolved VOCs.

To this end, we investigate the interactions of chromium (III) with volatile organic compounds (xylene, toluene, styrene, methyl ethyl ketone, carbon tetrafluoride, ascorbic acid, naphthalene, and anthracene) in a liquid layer of atmospheric particle. The total energy of the system and diffusion coefficients of each of these VOCs in the different environments were quantified and the molecular interactions were analyzed.

## Simulation methodology

In order to explore the physico-chemical interactions of Cr(III) with VOCs in the atmospheric conditions and its variation from the solution chemistry, several simulations were designed systematically. All atom molecular dynamic (MD) simulations were performed through GROMACS 2018.3. The actual atmospheric environment is very complex and in order to explore the effect of VOCs on Cr(III) containing particle, we need to include the effect of acidity, light intensities, and presence of other pollution gases such as NO_x_, SO_x_ and O_3_. However, the aim of our current is primarily to explore the segregated effect of VOCs. Several volatile organic compounds and poly aromatic compounds including xylene, toluene, styrene, methyl ethyl ketone, carbon tetrachloride, ascorbic acid, naphthalene, and anthracene were selected for the analysis. In general, there were 34 systems, which are shown in Table 1. The charge neutrality of the system was maintained by adding chloride ions.

**Table 1:** Details of the systems simulated with the number of molecules used.

	Designed systems	Cr(III)	VOCs	Water	Air
1	Reference	10	–	4035	–
2		10	–	4035	1000
3	Xylene	10	10	4035	–
4		10	10	4035	1000
5		0	10	4035	–
6		0	10	4035	1000
7	Toluene	10	10	4035	–
8		10	10	4035	1000
9		0	10	4035	–
10		0	10	4035	1000
11	Styrene	10	10	4035	–
12		10	10	4035	1000
13		0	10	4035	–
14		0	10	4035	1000
15	Methyl ethyl ketone	10	10	4035	–
16		10	10	4035	1000
17		0	10	4035	–
18		0	10	4035	1000
19	Carbon tetrachloride	10	10	4035	–
20		10	10	4035	1000
21		0	10	4035	–
22		0	10	4035	1000
23	Ascorbic acid	10	10	4035	–
24		10	10	4035	1000
25		0	10	4035	–
26		0	10	4035	1000
27	Naphthalene	10	10	4035	–
28		10	10	4035	1000
29		0	10	4035	–
30		0	10	4035	1000
31	Anthracene	10	10	4035	–
32		10	10	4035	1000
33		0	10	4035	–
34		0	10	4035	1000

In each designed system, water, Cr^3+^ ion, and either of the volatile organic compounds were inserted randomly in a box with size 5 × 5 × 5 nm^3^. Moreover, air (N_2_ and O_2_, ratio 78:22) was placed in alternate systems to explore and compare Cr(III)/VOC interactions in the atmosphere, than those within the solution environment. In particular, we chose to simulate the systems with ten molecules of Cr(III) and ten molecules of volatile organic compounds (~ 133 mM in water). The concentration is although high, but was chosen to get statistically precise results within reasonable computational time. We do note that because of having a high number of molecules placed in the box, the concentrations of some volatile organic compounds are higher than its solubility limits. Simulations were also performed without Cr(III) for the comparison.

The optimized geometries and force field parameters (bond constants, partial charges, angles, and dihedrals force constants) of air (nitrogen and oxygen) and all volatile organic compounds were obtained from Automated Topology Builder (ATB)^28^. All other parameters were obtained from the gromos54a7 set and were compatible with the standard gromacs forcefield. The Lennard–Jones (LJ) parameters (ε = 0.25 kcal/mol and r* = 0.112 nm) of Cr(III) were taken from Bronco et al.^29^. TIP4P model was selected for water, as it provides better results for gas–liquid equilibrium systems. The steepest descent minimization algorithm with a constraint of 1000 kJ/mol/nm maximum force was used for the energy minimization of the initial box. Periodic boundary conditions were applied in all three directions. Consequently, relaxation of the designed systems for temperature and pressure minimizations using NVT and NPT equilibrations were computed for 100 ps at 1 bar and 298 K. The temperature was kept constant through Berendsen thermostat and pressure was kept constant by Parrinello–Rahman methods^30,31^. Coordinate, energy, and velocity outputs were recorded every 10 ps. LINCS constraint algorithm was applied for all the bonds with fourth order. Moreover, a cut-off 1 nm was selected for LJ interactions and short-range Coulomb. Particle Mesh Ewald (PME) method with a fourth order and 0.16 nm grid spacing was used to calculate long-range electrostatic interactions. A long 10 ns production run at 298 K and 1 bar was performed. GROMACS package can provide an extensive library for the visualization and analysis of trajectory^32,33^, which was further used. The diffusion coefficients and radial distribution functions were obtained from the simulation results using GROMACS and Visual Molecular Dynamics (VMD). A sum of short-ranged LJ and Coulombic potentials was computed to get the interaction between the molecules, which we later use as interaction energies.

**Figure 1:**
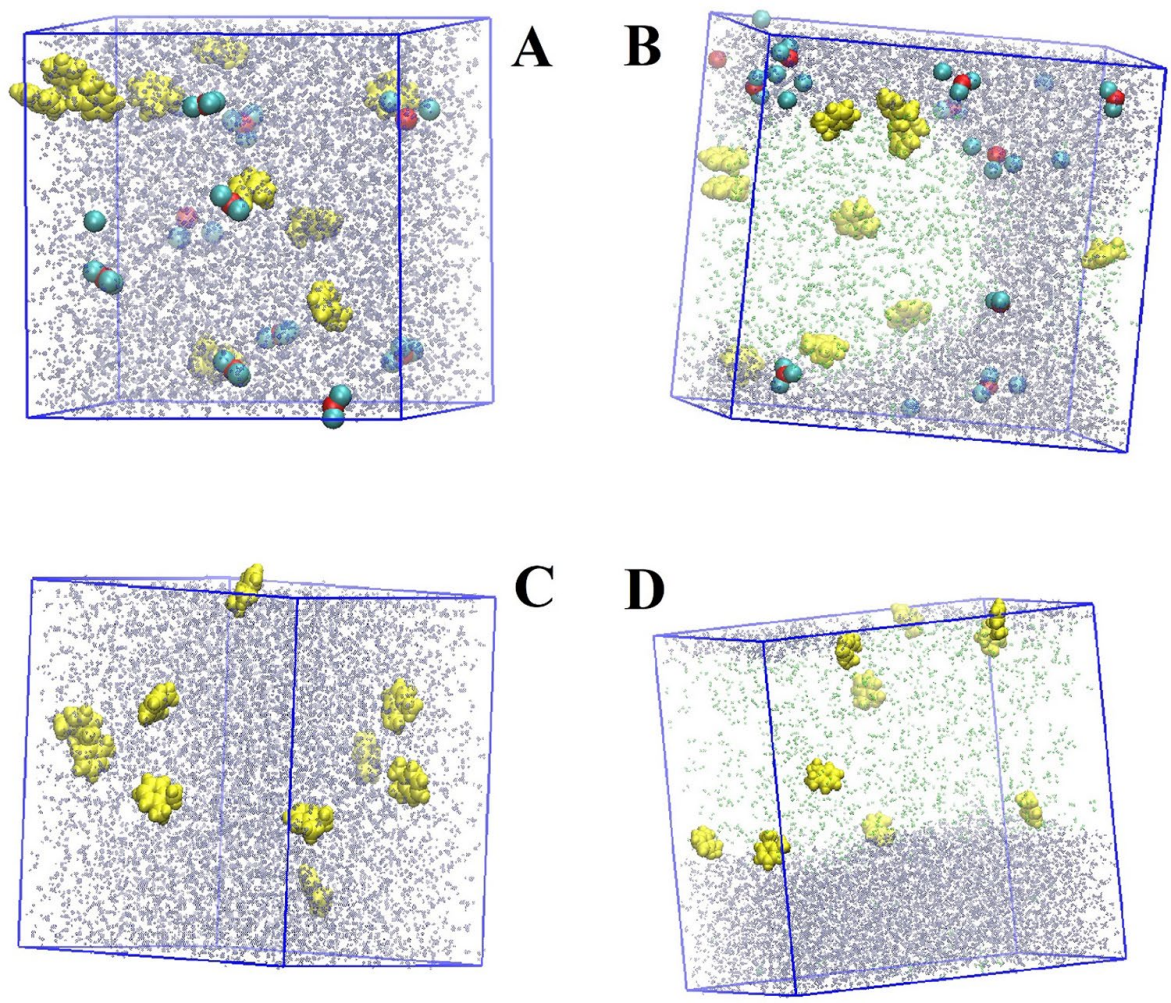
Illustrations of (**A**) System 3 with xylene (yellow) + Cr (red) with chlorine (cyan) + water (purple), (**B**) System 4 with xylene + Cr with chlorine + water + air (green), (**C**) System 5 with xylene + water, (**D**) System 6 with xylene + water + air after 10 ns of simulations.

Figure 1, presents a graphical view of designed systems, at the end of simulations, with xylene, for example. Systems with other VOCs were created and simulated in a similar way and figures are shown in the supplementary material (Figure S1). Figure 1A presents the end of the simulation of system 3, containing VOC and Cr(III) in water; whereas, Fig. 1B presents the end of the simulation of system 4, with VOC, Cr (III), water and air. Figure 1B clearly shows the interface between liquid and air phase, and based on their solubility, Cr(III) and xylene separate in different phases. In particular, xylene, being hydrophobic and insoluble in water, remains mostly in the gas phase or on the liquid–gas interface. Cr(III), on the other hand, along with the Cl ions stays in the liquid phase. Figure 1C,D are snapshot from systems 5 and 6, which are equivalent to system 3 and 4, respectively, except without Cr(III). While the simulations results (snapshot as seen in Fig. 1B) are not equivalent to spherical aerosol particles, as observed in atmosphere; it mimics the interfacial and surface interactions and in that sense provides insights to the intermolecular interactions occurring within atmospheric particles. Hence, we refer to it as aerosol/particulate throughout the work. An important note herein is that simulations were performed with periodic boundary conditions.

## Results and discussion

### Validation of force-field parameters

The validation of the simulation parameters is of critical importance before exploring Cr(III)/VOC interactions. Hence, we begin with validating results for pure component simulations. All of the simulations reached equilibrium within the first ns of the production run. For analysis, the last 2 ns of the long 10 ns run was used and averaged over. Molecular simulation of aqueous CrCl_3_ resulted in Cr(III) coordination number of 5.5, which is in close agreement with a value of 5.7 reported in the literautre^29^. Furthermore, MD simulations were performed on xylene, toluene, styrene, methyl ethyl ketone, carbon tetrachloride, ascorbic acid, naphthalene, and anthracene, individually. The theoretically computed density, for example, of ascorbic acid was obtained to be 1.554 ± 0.0034 g/cm^3^, with the reported experimental value of 1.650 g/cm^3^^34^. Moreover, the self-diffusion coefficient of ascorbic acid was determined to be 7.247 × 10^−5^ cm^2^/s, while the literature value is 1.933 × 10^−5^ cm^2^/s^35^. Likewise, Table 2 reports density and self-diffusion coefficient of all of the VOCs (except anthracene and naphthalene, which are solid at room conditions). The results from the simulations of the individual components are within limits of the reported values, supporting the choice of the forcefield parameters. While the simulation values of diffusion coefficient differ significantly for certain components like styrene, the deviations are expected for viscous components due to sluggish dynamics, as also noted in the literature^27^ In the next section, results from the simulations of aqueous solutions of VOCs with and without CrCl_3_ and air are analyzed.

**Table 2:** Computed densities and self-diffusion coefficients of the pure compounds as obtained from the simulations and their comparison with the literature at 298 K and 1 bar. The standard deviation in the simulation data was less than 1%.

	Density (g/cm^3^)		Diffusion coefficients (cm^2^/s)	
	Computed	Literature	Computed	Literature
Xylene	0.880	0.861^36^	1.861 × 10^−5^	1.480 × 10^−5^^36^
Toluene	0.878	0.866^37^	2.308 × 10^−5^	1.810 × 10^−5^^37^
Styrene	0.906	0.901^38^	1.697 × 10^−5^	0.800 × 10^−5^^38^
Methyl ethyl ketone	0.776	0.805^39^	2.948 × 10^−5^	2.850 × 10^−5^^39^
Carbon tetrachloride	1.819	1.500^40^	1.375 × 10^−5^	2.170 × 10^−5^^40^

### Interaction energies of Cr(III) with VOCs

As noted above, MD simulations were performed to investigate the molecular interactions within Cr(III) containing atmospheric particles in the presence of different volatile organic compounds. We begin the analysis by examining the interaction energies between the components. In particular, the interactions of Cr(III) ion with volatile organic compounds including xylene, toluene, styrene, methyl ethyl ketone, carbon tetrachloride, ascorbic acid, naphthalene, anthracene with different scenarios of water and air were evaluated. The interaction energies were computed as the sum of short range LJ and columbic interactions between the molecules. The energies reported are the average values over the last 2 ns of the production run. The standard deviation for all obtained data was less than 1%. Table 3 reports energies between different components for all thirty four systems.

**Table 3:** Interaction energies, computed as the sum of short-range LJ and columbic interactions, for each system in kJ/mol at 298 K and 1 bar. Standard deviation was less than 1% in the data.

	System	Cr^+3^/water	Water/water	Cr(III)/additive	Additive/water	Additive/additive
1	Reference	− 11,583.42	− 162,249.01	–	–	
2		− 12,235.77	− 156,608.83			
3	Xylene	− 7539.44	− 163,316.43	3.63	− 584.83	1197.12
4		− 13,421.00	− 219,740.20	0.00	− 137.95	1162.29
5		–	− 229,130.02	–	− 617.67	1201.18
6		–	− 223,810.87	–	− 129.10	1192.13
7	Toluene	− 13,196.20	− 161,201.00	3.15	− 555.49	− 1652.41
8		− 14,770.48	− 155,619.93	0.18	− 126.76	− 1649.84
9		–	− 166,209.09	–	− 554.45	− 1651.75
10		−	− 161,826.46	–	− 58.99	− 1653.23
11	Styrene	− 9360.54	− 162,455.95	8.49	− 613.39	− 522.94
12		− 14,420.12	− 155,942.90	0.25	− 183.40	− 489.72
13		–	− 166,564.34	–	− 647.02	− 485.68
14		–	− 161,474.08	–	− 214.51	− 493.14
15	Methyl ethyl ketone	− 12,974.23	− 161,673.67	− 3.38	− 659.32	− 974.32
16		− 11,051.17	− 157,724.05	0.03	− 411.59	− 960.74
17		–	− 166,469.61	–	− 700.90	− 957.48
18		–	− 161,524.57	–	− 409.20	− 962.50
19	Carbon tetrachloride	− 11,708.44	− 161,323.78	2.75	− 422.11	− 24.02
20		− 11,492.36	− 157,566.59	2.72	− 67.86	− 15.07
21		–	− 166,912.19	–	− 433.06	− 15.14
22		–	− 162,016.05	–	− 71.97	− 35.43
23	Ascorbic acid	− 14,539.73	− 160,170.88	61.45	− 2593.28	− 4163.06
24		− 13,783.17	− 156,261.14	− 688.79	− 2223.30	− 4063.90
25		–	− 166,334.75	–	− 2762.60	− 4100.70
26		–	− 160,746.14	–	− 2473.48	− 4006.11
27	Naphthalene	− 6097.73	− 163,521.75	12.61	− 738.42	− 478.96
28		− 5934.08	− 157,838.24	6.03	− 279.46	− 279.45
29		–	− 165,952.86	–	− 717.60	− 493.98
30		–	− 160,033.28	–	− 313.51	− 465.27
31	Anthracene	− 9900.80	− 162,740.51	2.59	− 603.99	− 1442.43
32		− 22,034.85	− 159,881.37	6.41	− 317.33	− 1202.52
33		–	− 166,557.46	–	− 652.22	− 1412.99
34		–	− 160,301.32	–	− 350.97	− 1210.70

#### Solution behavior differs from aerosol

Comparisons between the interaction energies in the presence and absence of air showed that air presence directly affects the interaction energies of Cr(III)/water and water/water case. For instance, by comparing systems 1 and 2, we observed that the interactions between water molecules decreased from − 162,249.01 to  − 156,608.83 kJ/mol, while Cr(III)/water interaction energy increased from − 11,583.42 to  − 12,235.77 kJ/mol. A difference in the energies, in the presence of air, compared to the systems without air, was observed for all the systems. The interactions between water molecules decreased for all the systems (in all VOCs) without Cr(III) in the presence of air, which is due to the absorption of nitrogen and oxygen molecules in water. The same was also valid for systems with Cr(III) and VOCs, except for the case with xylene, wherein the interactions between water increased by 86% in the presence of air. This increase could be due to the fact that xylene is practically insoluble in water, as seen in Fig. 1. The simulations, as mentioned above, were carried with 10 molecules of VOCs, irrespective of their solubility in water. The presence of air assists in improving water/water interaction in the presence of xylene, for the reasons mentioned above.

Further, the interactions between Cr(III) and water also was affected in the presence of air. While the presence of air enhanced Cr(III)/water interactions in the reference case, and in the presence of xylene, toluene, styrene, and anthracene, it decreased in the presence of methyl ethyl ketone, carbon tetrachloride, ascorbic acid, and naphthalene. In particular, a strong increase, ~ 122%, was observed in the presence of anthracene, and a strong decrease, ~ 54%, was noted for styrene. An explanation of the above-mentioned phenomena will be explained later. However, taken together, the results indicate that the experimental data observed from the chromium solutions cannot be extended to the aerosol systems, wherein intermolecular interactions, solubility, kinetics, diffusion, and other transport, thermophysical, and chemical properties can be different.

#### VOCs affect chromium behavior within the particle

The interactions within the aerosols were investigated. Interestingly, there are extremely weak to no interactions between Cr(III) and additives (xylene, toluene, styrene, methyl ethyl ketone, carbon tetrachloride, naphthalene and anthracene) as noted in Table 3. As most of the additives used in the simulations are insoluble in water and Cr(III) has strong interactions with water, the low to none interactions observed between Cr(III) and additives is inferential. However, we did observe a strong interaction between Cr(III) and ascorbic acid as the interaction energies. Gu et al.^41^ analyzed the reduction of hexavalent chromium to trivalent chromium by ascorbic acid in aqueous solution and showed that ascorbic acid preferentially favored reduction of Cr(VI) to Cr (III). These findings were in good agreement with our studies, as we, too, observe strong interactions between Cr(III) and ascorbic acid was greater in comparison to others. For example, the energy was 61.45 kJ/mol (in the absence of air), and it decreased to − 688.78 kJ/mol (in the presence of air). The observed behavior could be due to the extremely high solubility of ascorbic acid in water, as also seen through ascorbic acid/water interactions. The additive/additive interactions also followed a similar trend (consistent with the solubility of the VOCs in water) with ascorbic acid having the highest interactions and xylene having the weakest.

Further, we explored the effect of these additives on Cr(III) interactions and vice-versa. While Cr(III) does not have any direct interactions with the additives, the additives did affect the Cr(III)/water interactions. The reference case energy between Cr(III)/water is − 12,235.77 kJ/mol in the presence of air. The interaction energy is slightly increased in the presence of xylene, toluene, styrene, and ascorbic acid. Moreover, the interactions slightly decreased in the presence of methyl ethyl ketone and carbon tetrachloride. On the other hand, a strong decrease in the interaction was observed for naphthalene. In the system with anthracene, which is similar in structure to naphthalene, the energy, interestingly, increased by ~ 80%. The results suggest that the effect of VOCs on chromium/water interactions strongly depends on the type of the VOCs.

#### Diffusion within the particles

The diffusion coefficient was computed using the Einstein–Smoluchowski equation ($< x >^{2} = 2Dt$), wherein $< x >^{{}}$ is the mean squared deviation, as computed from the last 2 ns of the trajectory, *t* is time, and D is the diffusion coefficient. Diffusion coefficients of the additives for all systems are reported in Table 4. The standard deviation in the reported data was less than 5% for all the cases. As seen from Table 4, the diffusivity values of all the volatile organic compounds were between 1 × 10^−5^ cm^2^/s and 16 × 10^−5^ cm^2^/s in systems 3 to 34. Moreover, the diffusivity coefficients were higher in the presence of air and Cr(III). The diffusion coefficients of Cr were close to previous studies^27^, which is equal to 3 × 10^−5^ cm^2^/s–8 × 10^−5^ cm^2^/s. This observation implied that the transfer of Cr(III) and volatile organic compounds in a typical 50 nm PM would occur from few microseconds to nanoseconds.

**Table 4:** Diffusion coefficients (D) of the additives in the aerosols for all simulated systems.

	System no	D (× 10^−5^ cm^2^/s)	System no		D (× 10^−5^ cm^2^/s)
3	Xylene	0.9475	19	Carbon Tetrachloride	1.8686
4		4.7166	20		7.6835
5		0.9376	21		2.2226
6		8.8610	22		15.5783
7	Toluene	1.5856	23	Ascorbic acid	1.4385
8		13.1978	24		2.0862
9		1.1719	25		0.6135
10		13.0362	26		1.1944
11	Styrene	1.9867	27	Naphthalene	0.9715
12		16.6546	28		5.6786
13		1.9588	29		1.1168
14		11.8561	30		10.7646
15	Methyl ethyl ketone	1.9026	31	Anthracene	0.8332
16		5.1107	32		7.3416
17		3.0317	33		1.1888
18		5.8349	34		3.4650

## Conclusion

The results of the molecular dynamics simulation study demonstrated the Cr interactions with volatile organic compounds in the deliquesced particle. The volatile organic compounds, Cr, and water were considered as particle matrix. The results of the simulation propose that the presence of air influences interactions between the molecules in the atmospheric droplet. Chromium did not interact with xylene, toluene, styrene, methyl ethyl ketone, carbon tetrachloride, naphthalene and anthracene, but it had strong interactions with ascorbic acid. The results also indicate that although VOCs might not directly impact Cr chemistry within the particles, but might affect the apparent kinetics within, due to the modified diffusion coefficient and due to the change of water structure around chromium.

## Supplementary information

Supplementary Information
