# Supplementary material for: Impact of volatile organic compounds on chromium containing atmospheric particulate: insights from molecular dynamics simulations
Source: Sci Rep. 2020 Oct 15;10:17387. doi: 10.1038/s41598-020-74522-x (PMC7567111; doi:10.1038/s41598-020-74522-x)
Supplement: Supplementary file 1 — Supplementary Information [file 41598_2020_74522_MOESM1_ESM.docx]

**Supplementary Material**

*Impact of volatile organic compounds on chromium containing atmospheric particulate: Insights from molecular dynamics simulations*

Dhawal Shah^1^, Mirat Karibayev^1^, Enoch Kwasi Adotey^1^, and Mehdi Amouei Torkmahalleh^1*^

| A)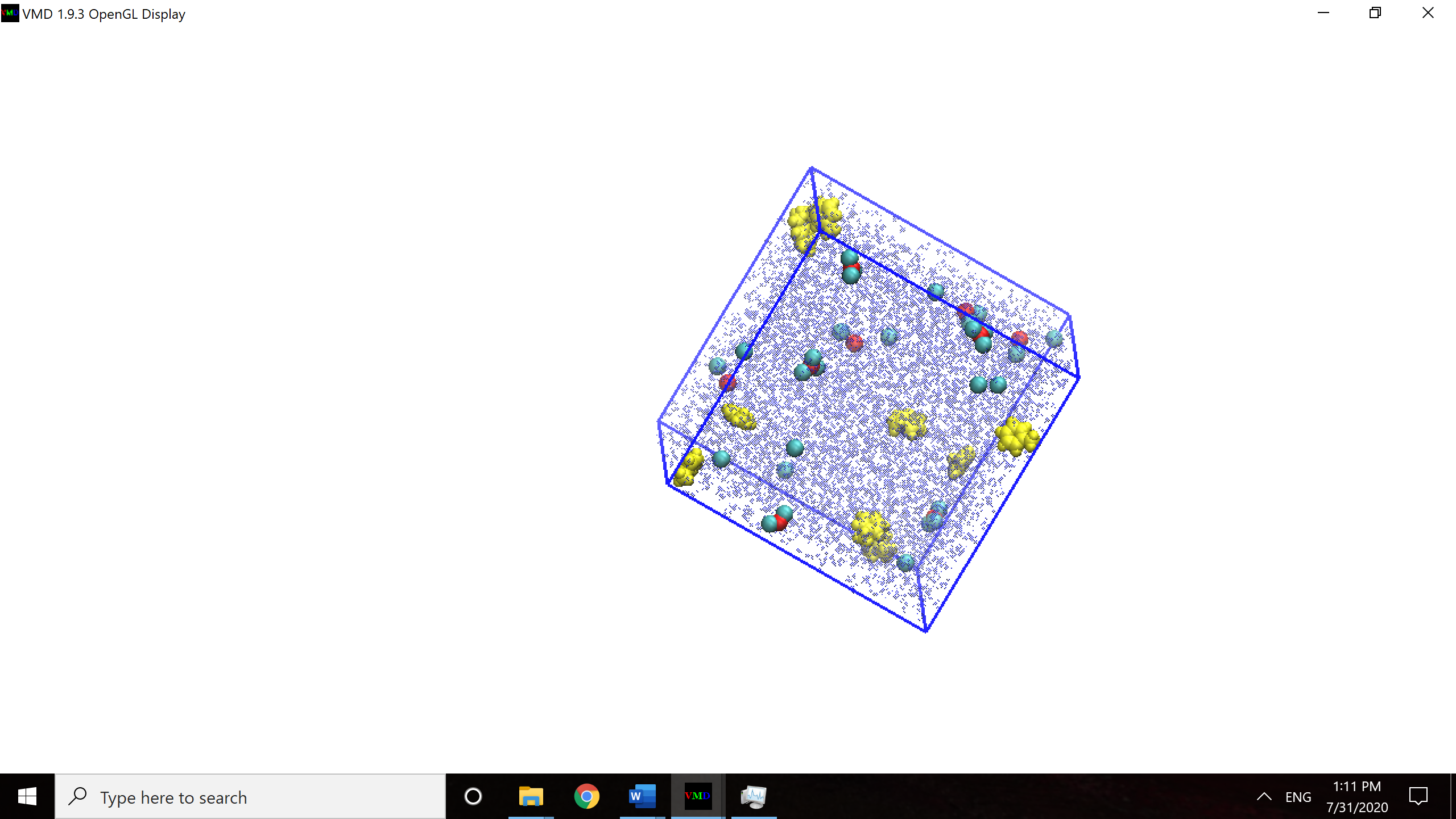 | B)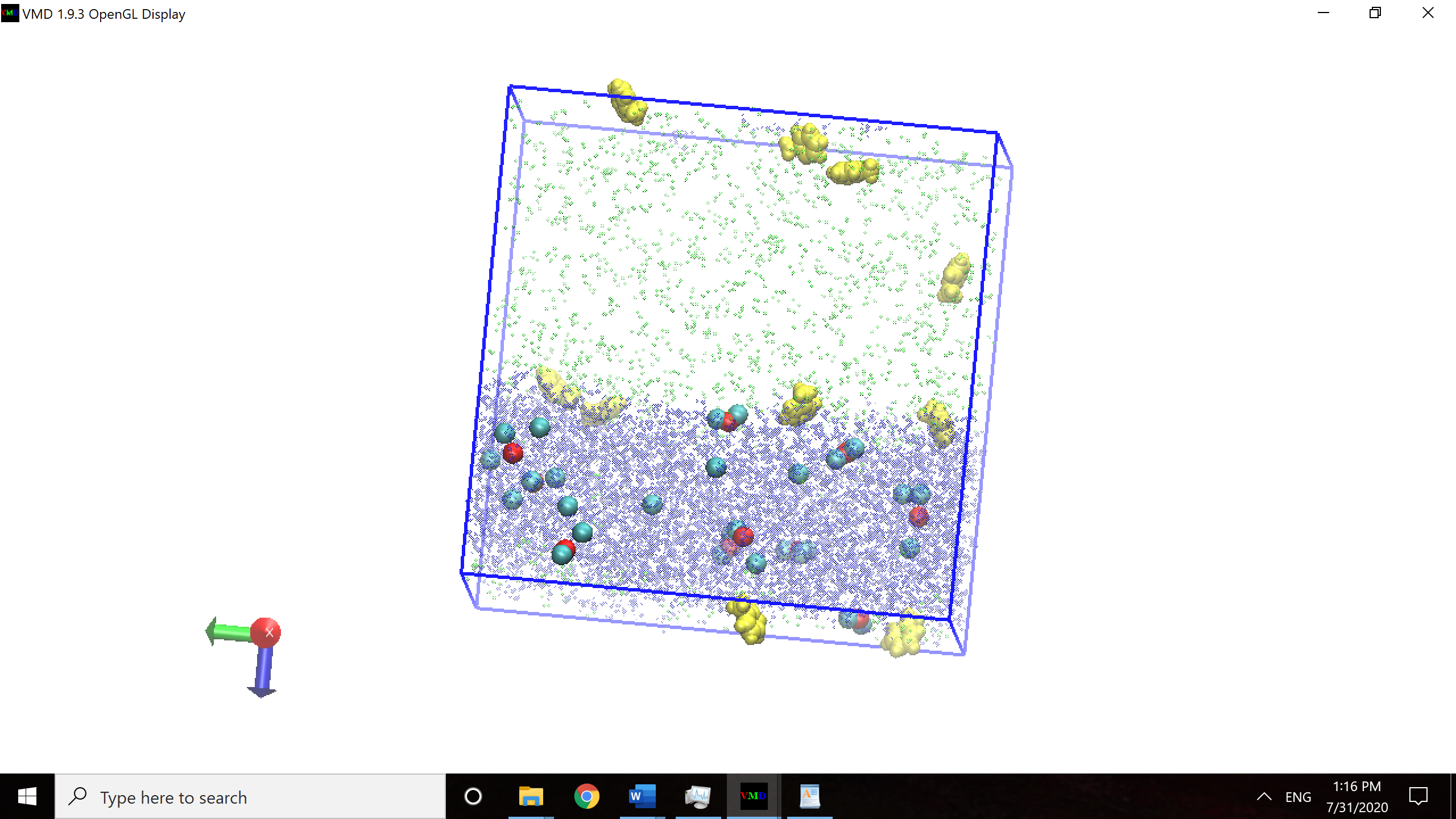 |
| --- | --- |
| 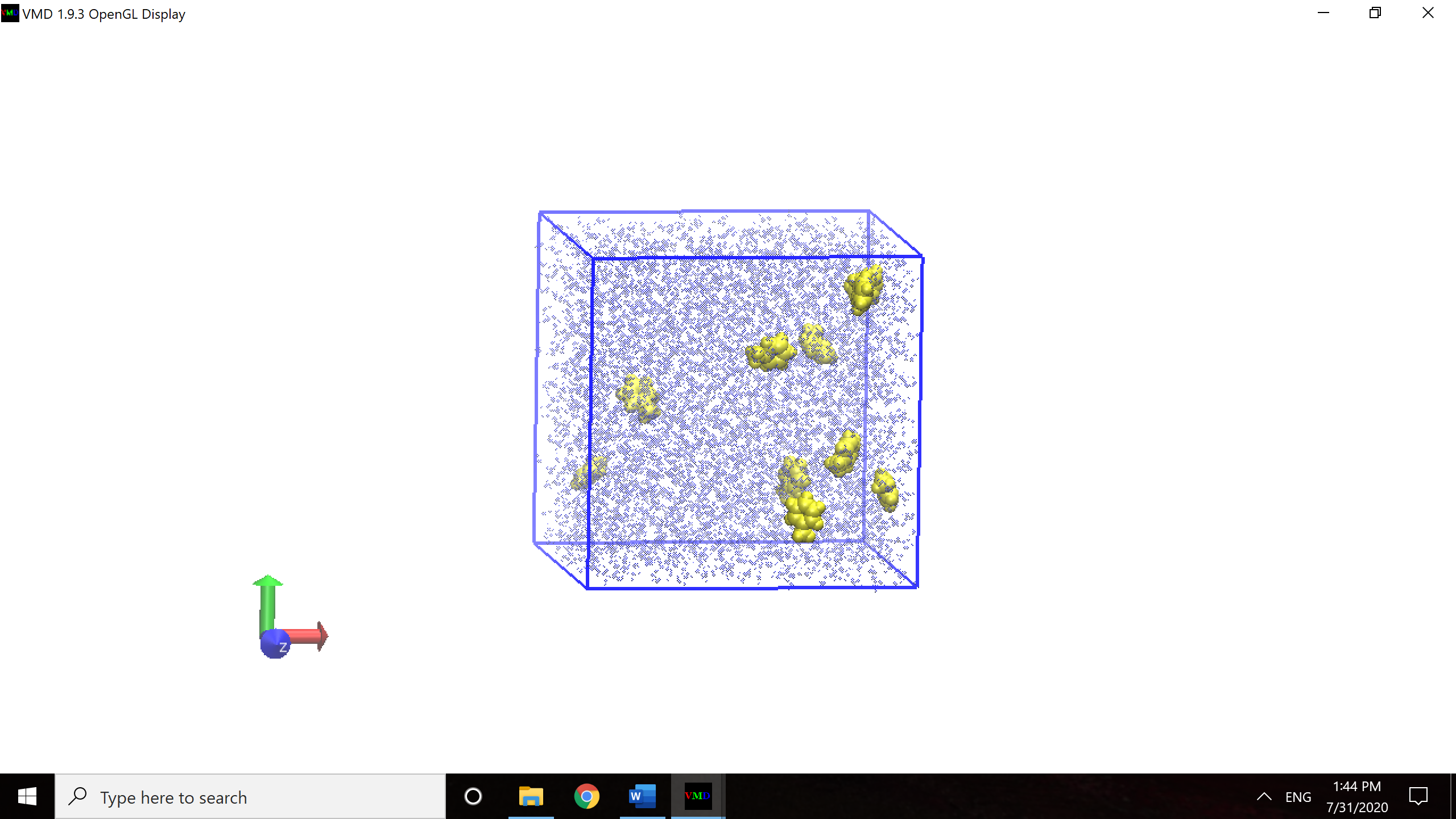  C) | 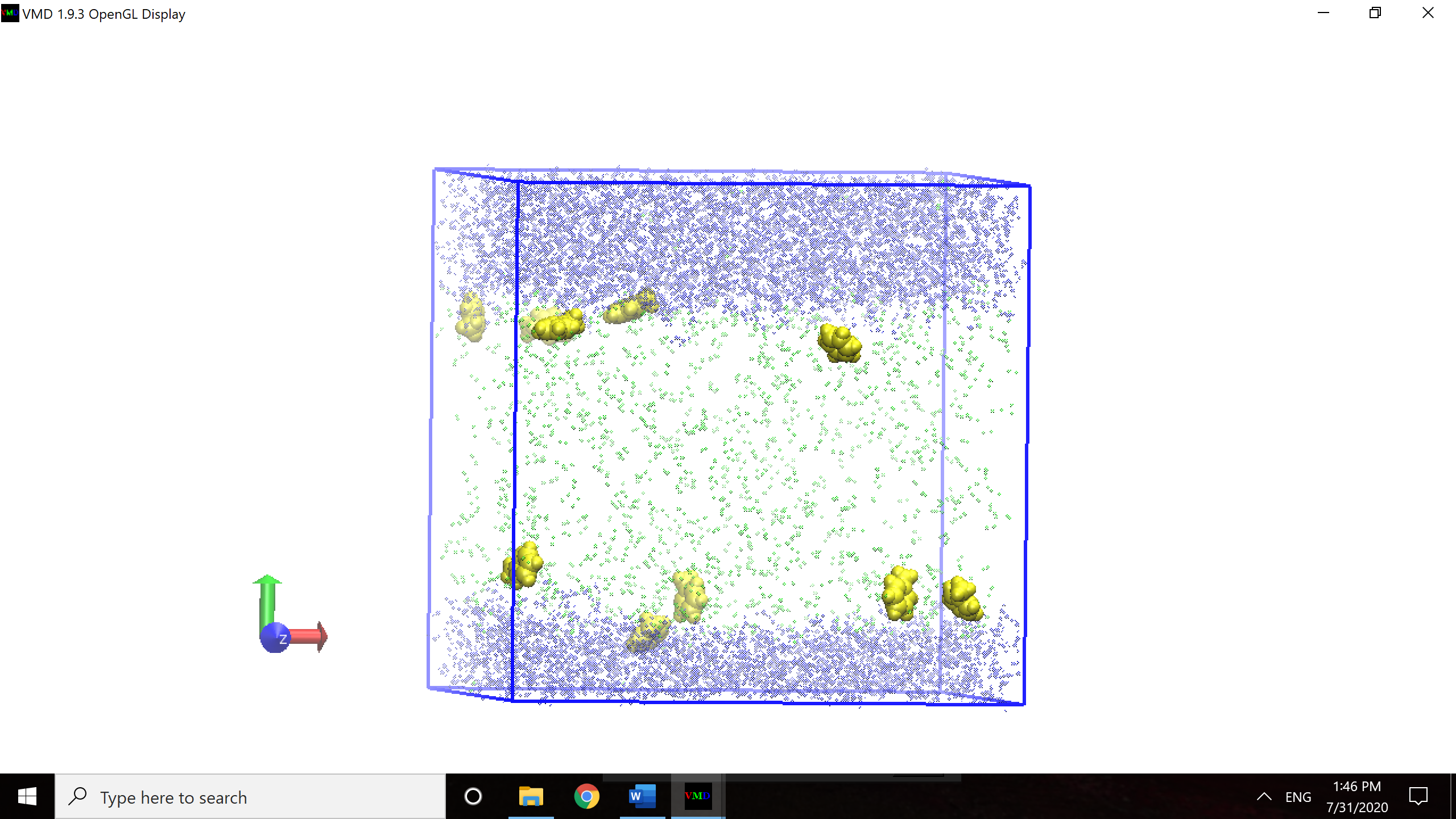  D) |

**Figure S1.1:** Illustrations of (A) System 7 with toluene (yellow) + Cr (red) with chlorine (cyan) + water (purple), (B) System 8 with toluene + Cr with chlorine + water + air (green), (C) System 9 with toluene + water, (D) System 10 with toluene + water +air after 10 ns of simulations.

| 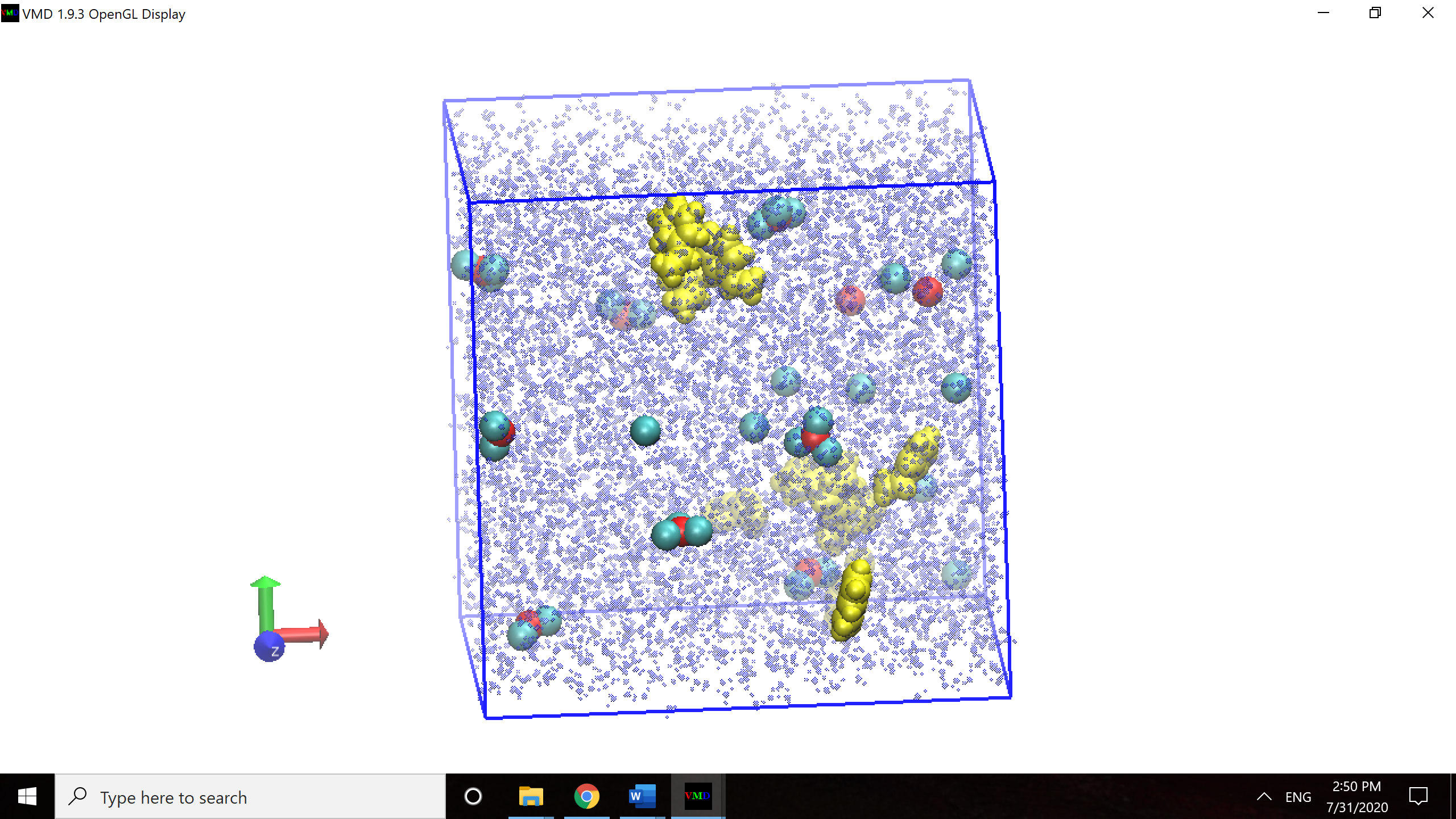  A) | 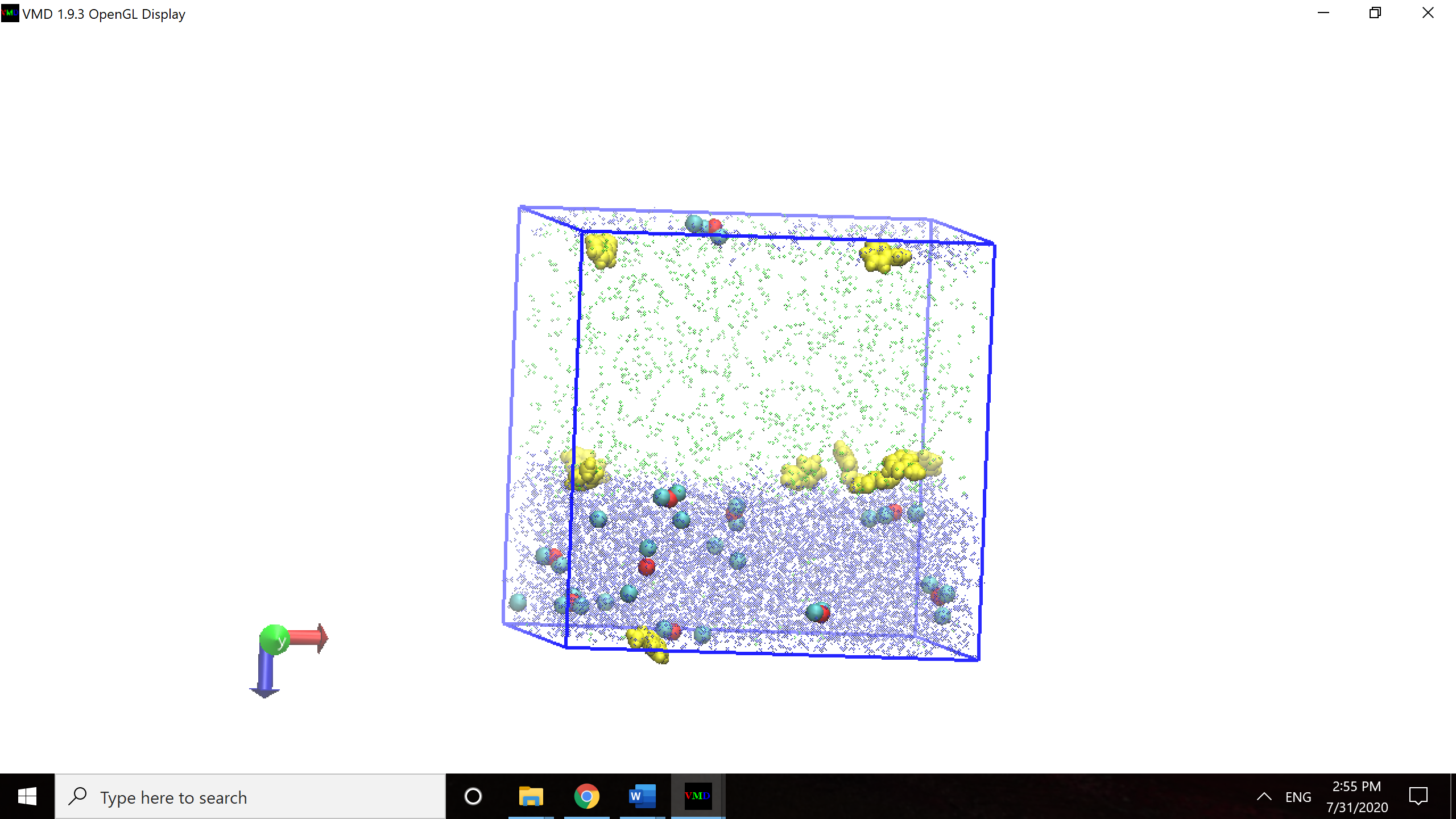  B) |
| --- | --- |
| 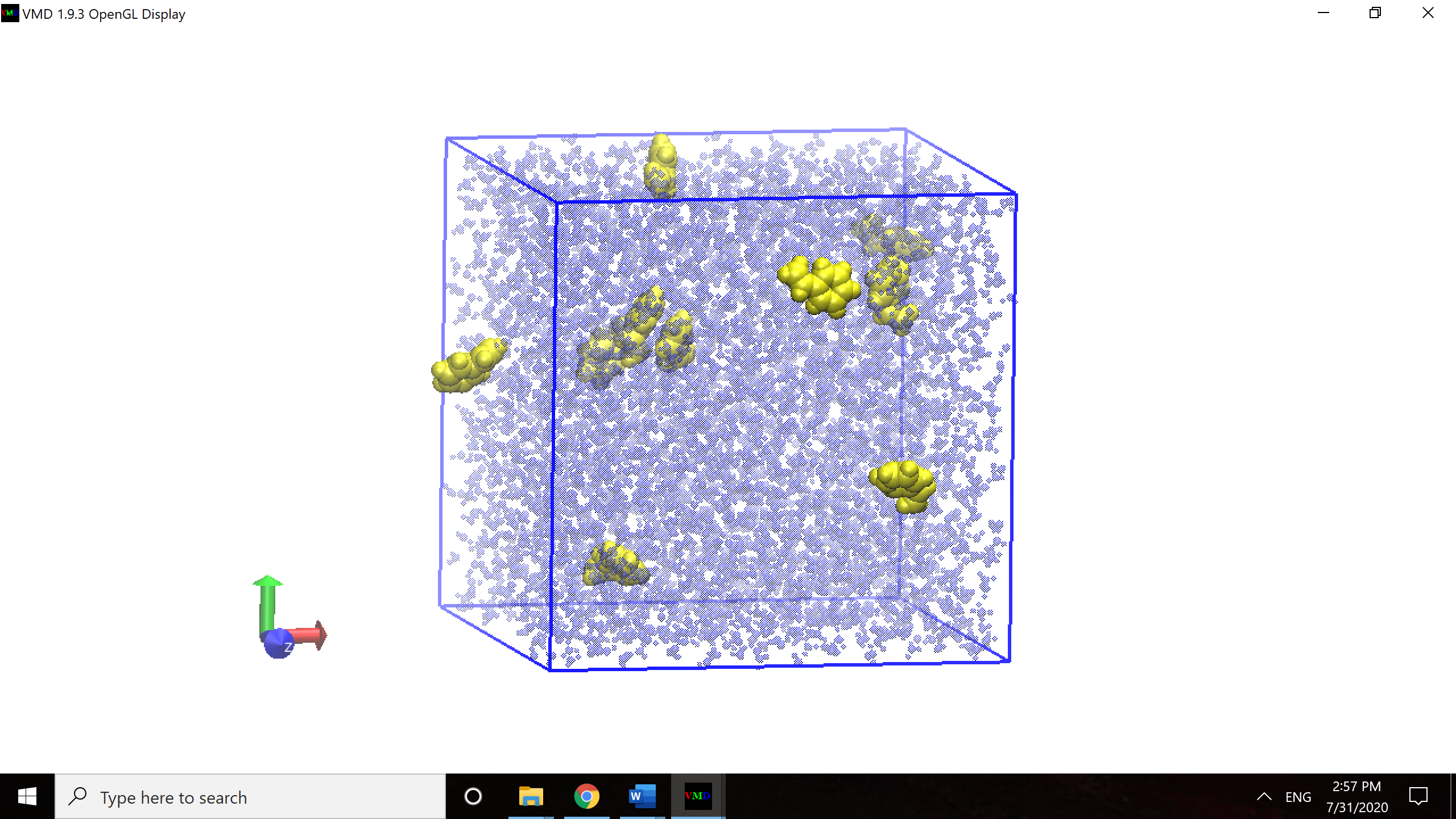  C) | 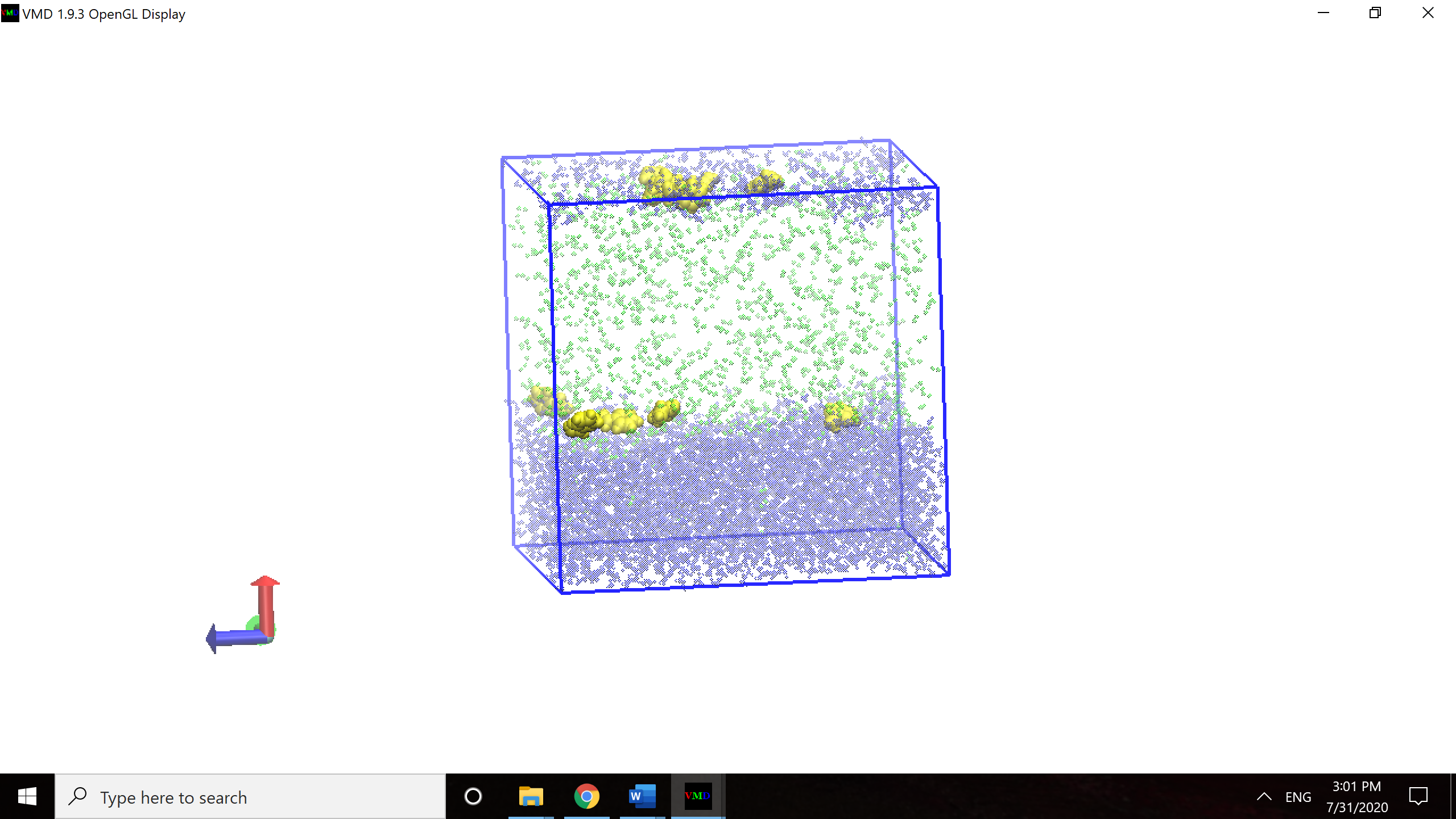  D) |

**Figure S1.2:** Illustrations of (A) System 11 with styrene (yellow) + Cr (red) with chlorine (cyan) + water (purple), (B) System 12 with styrene + Cr with chlorine + water + air (green), (C) System 13 with styrene + water, (D) System 14 with styrene + water +air after 10 ns of simulations.

| 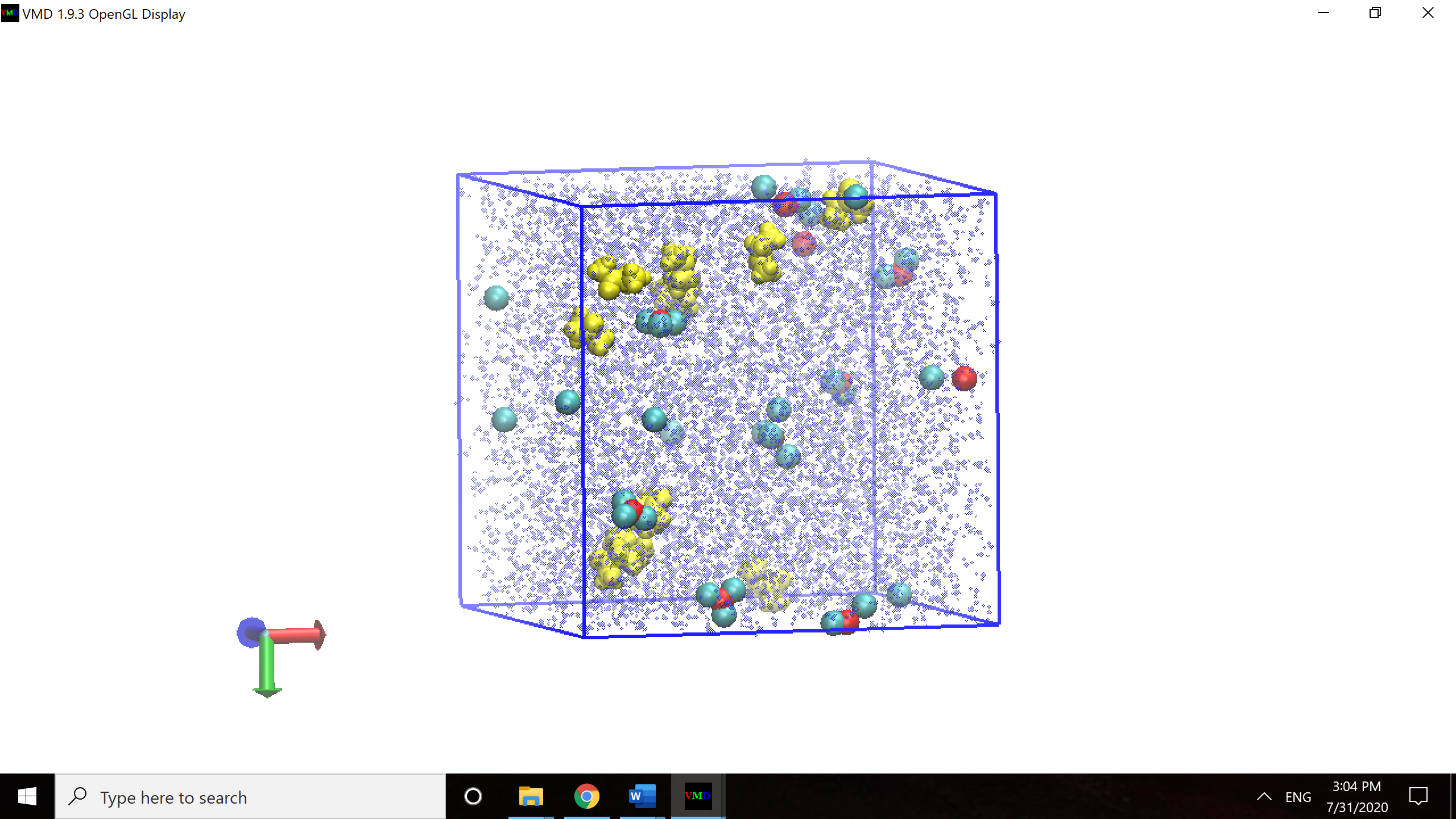  A) | 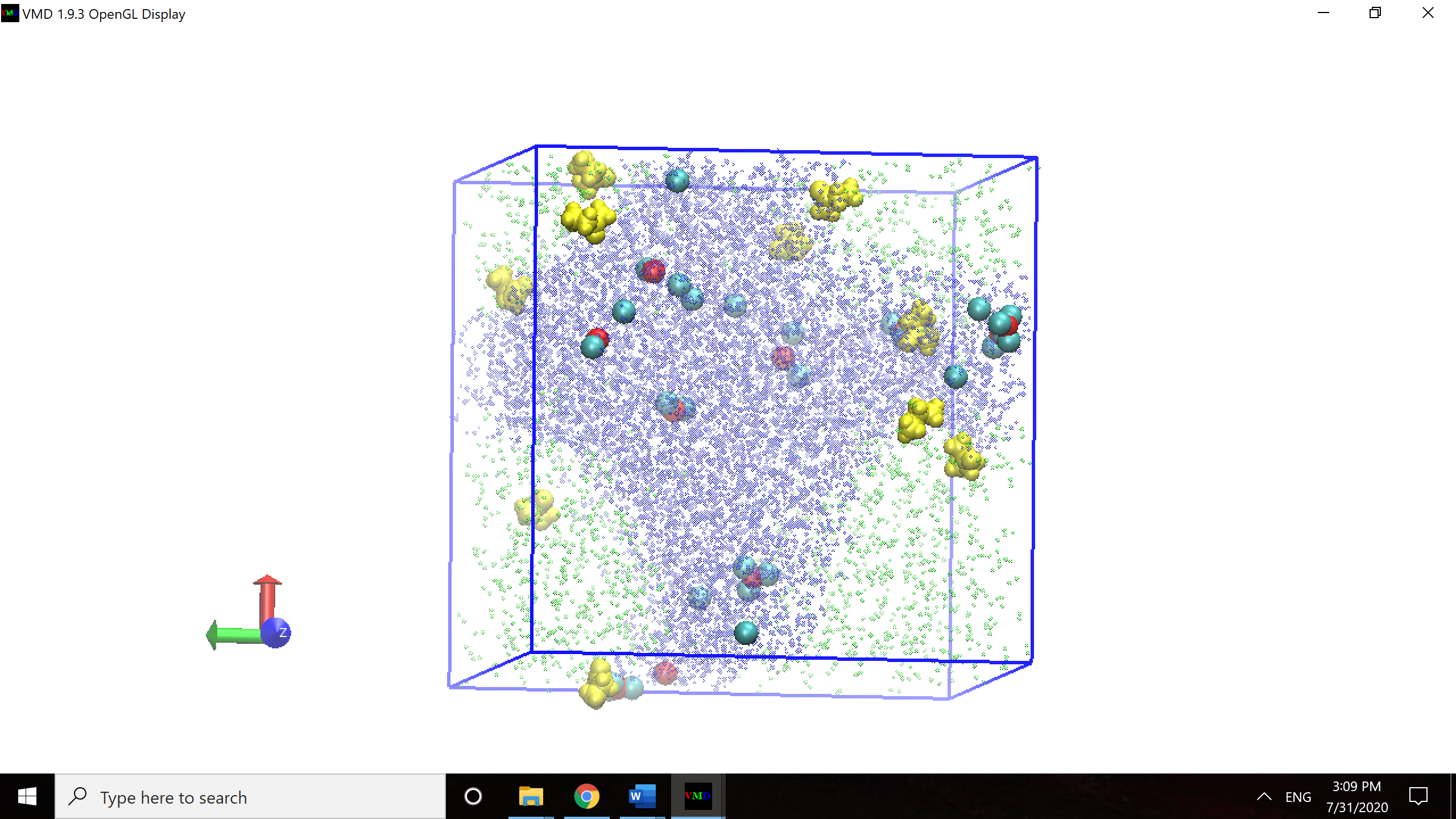  B) |
| --- | --- |
| 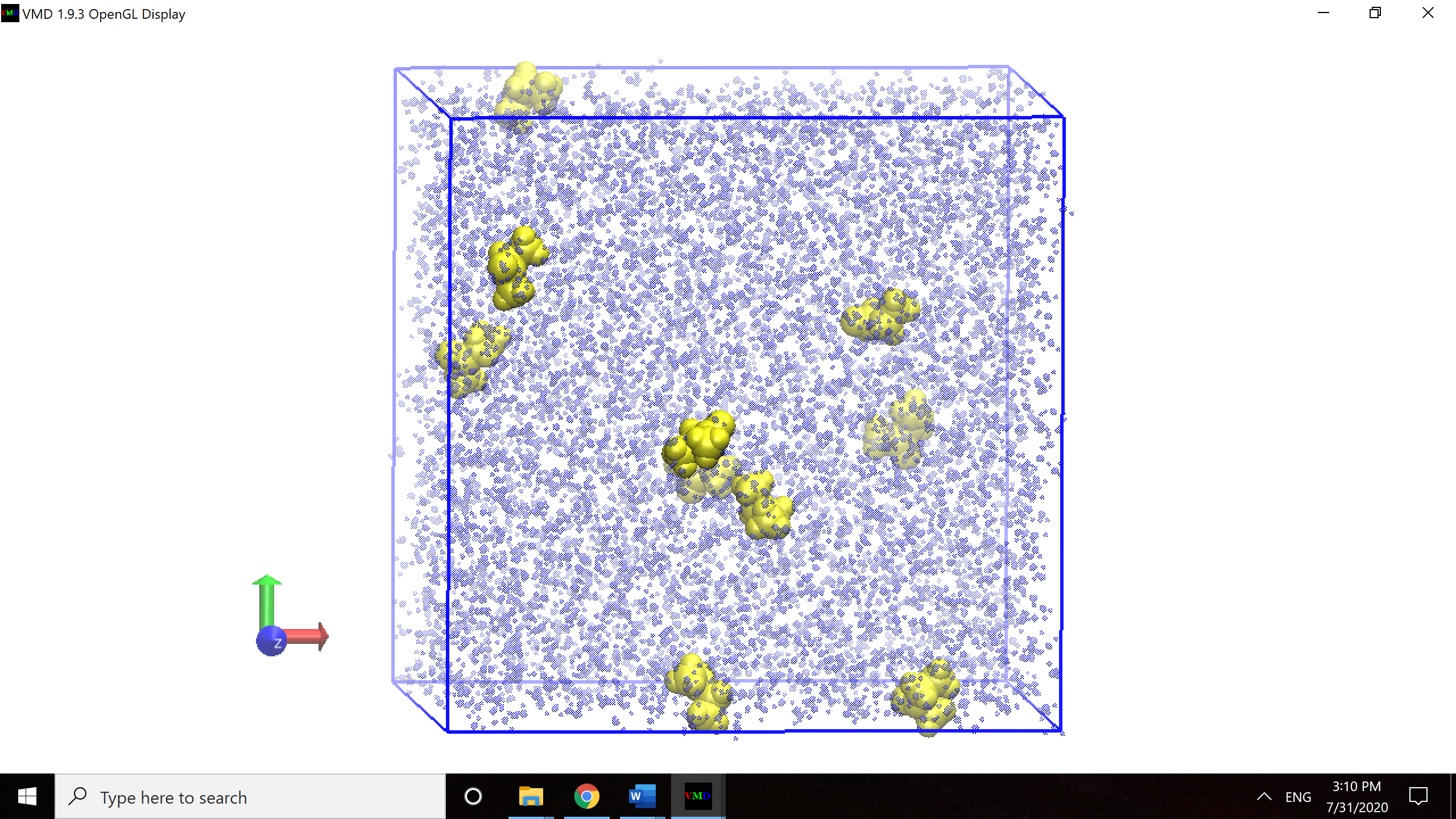  C) | 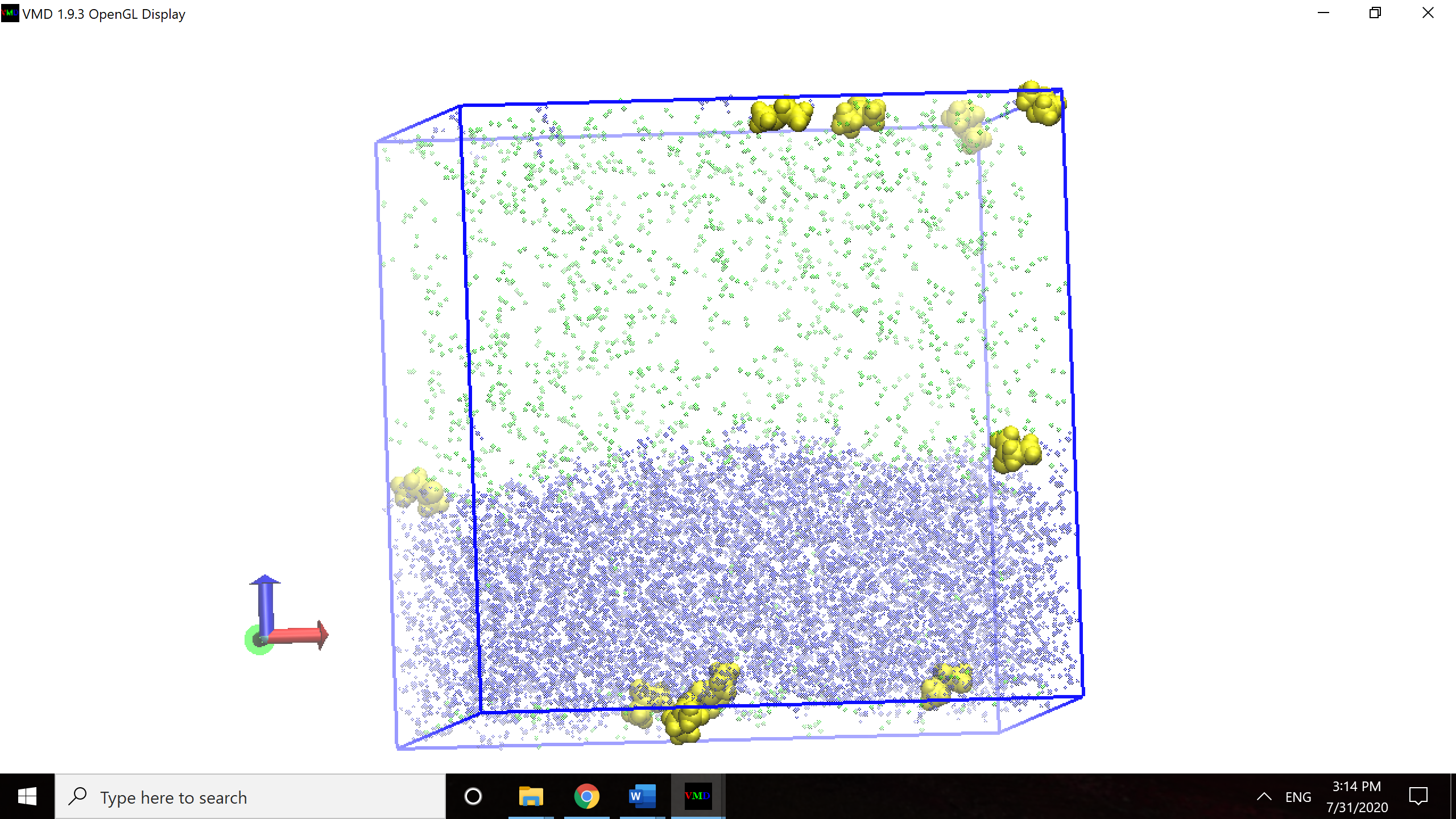  D) |

**Figure S1.3:** Illustrations of (A) System 15 with methyl ethyl ketone (yellow) + Cr (red) with chlorine (cyan) + water (purple), (B) System 16 with methyl ethyl ketone + Cr with chlorine + water + air (green), (C) System 17 with methyl ethyl ketone + water, (D) System 18 with methyl ethyl ketone + water +air after 10 ns of simulations.

| 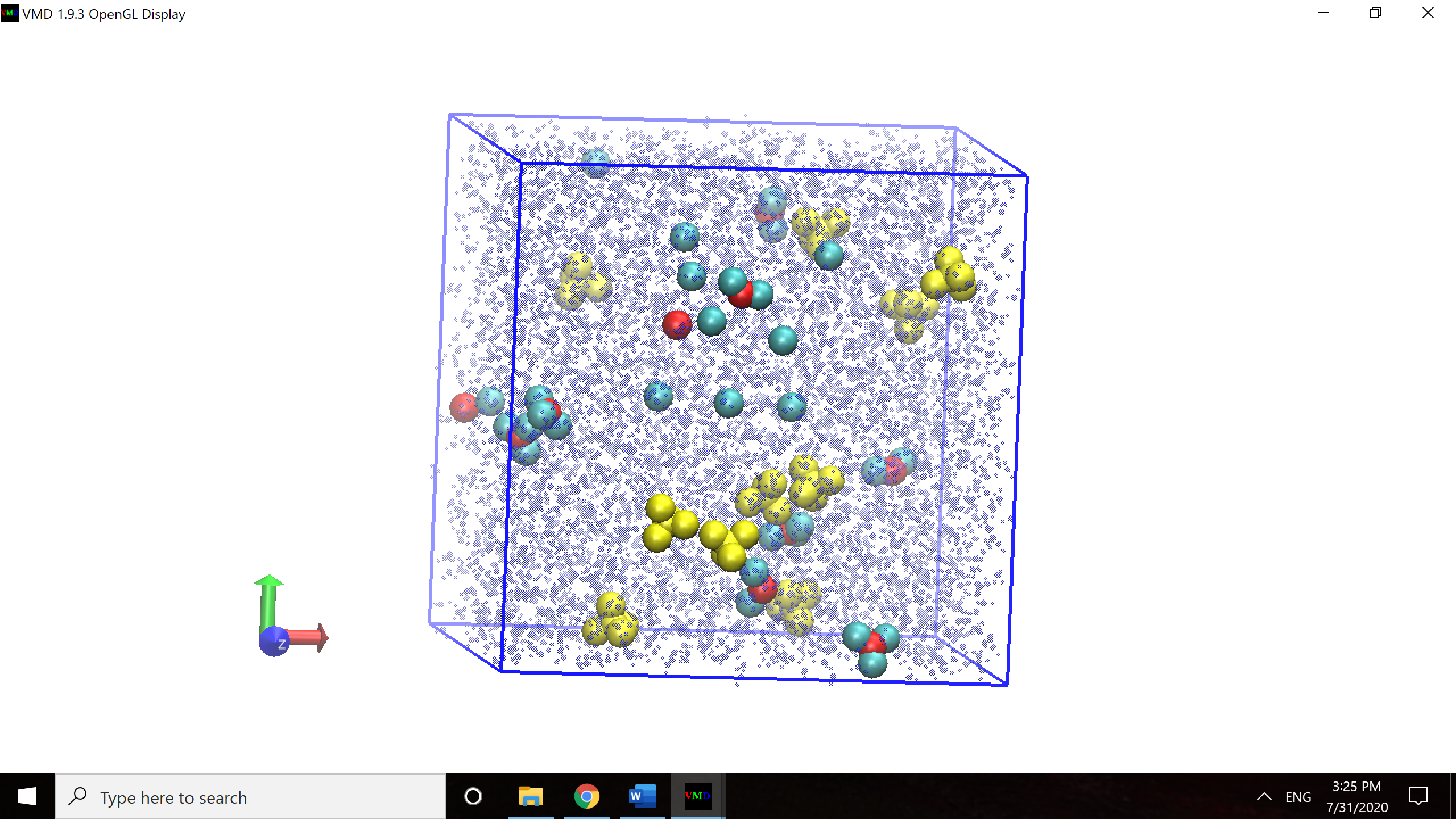  A) | 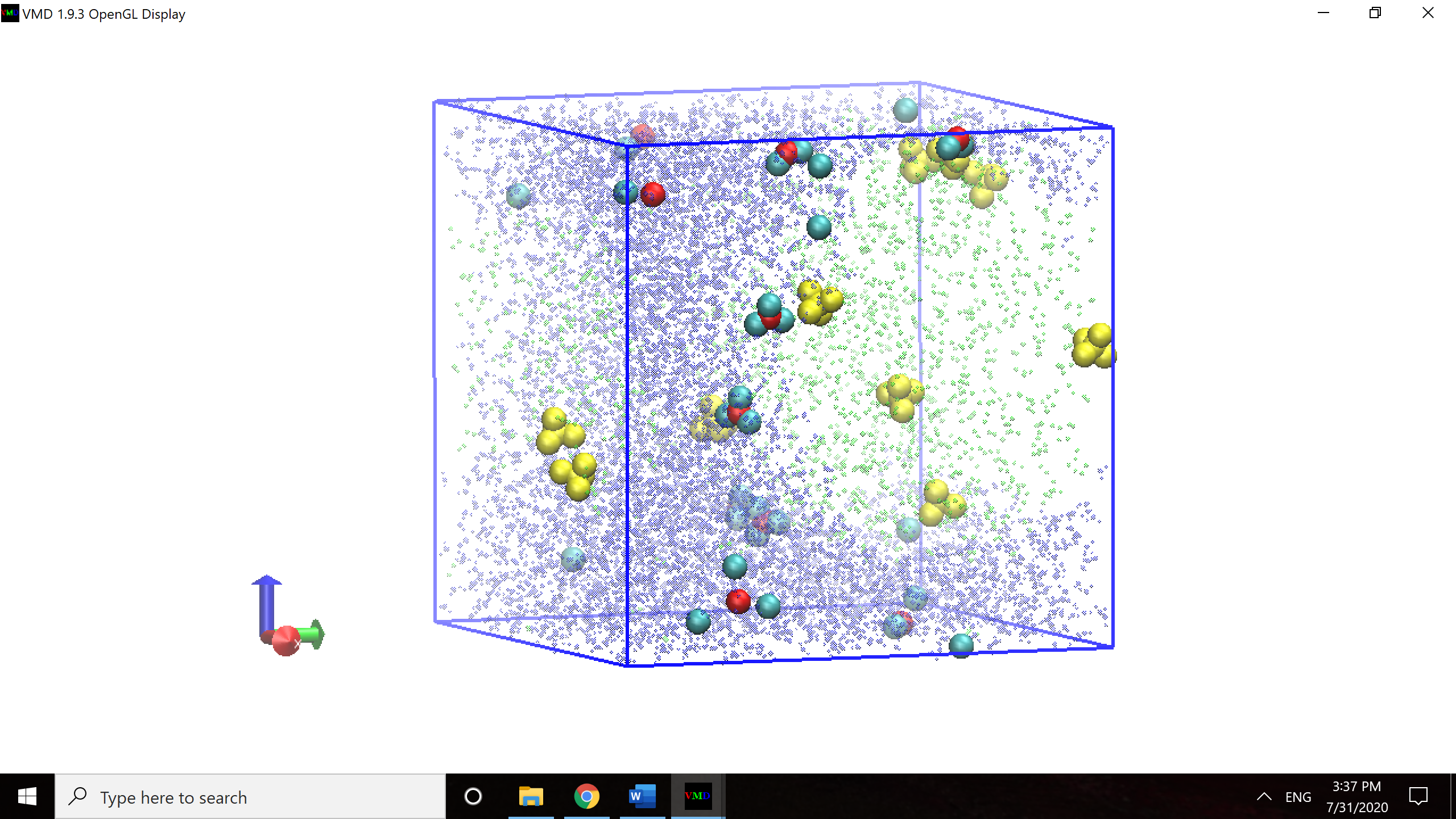  B) |
| --- | --- |
| 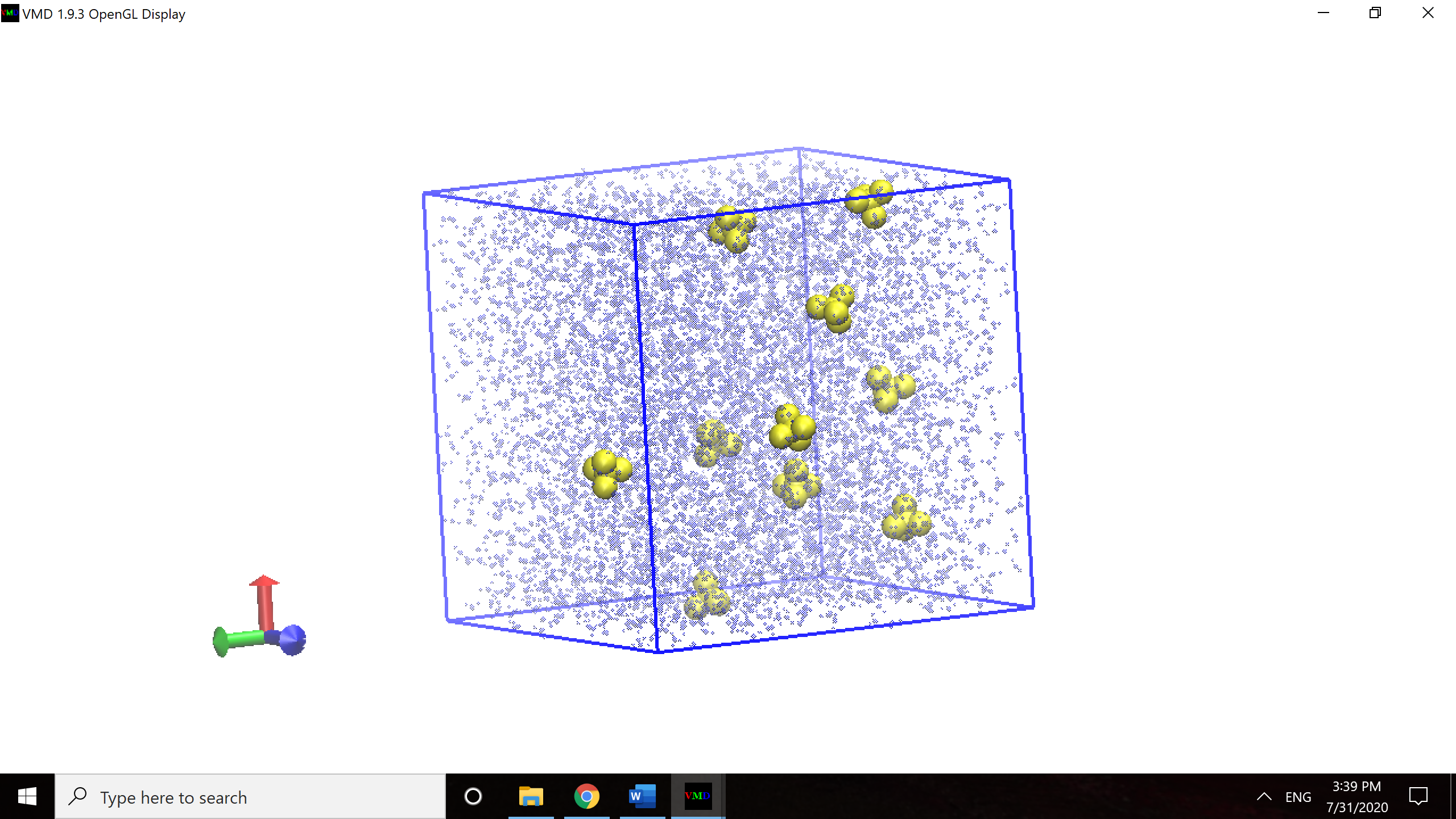  C) | 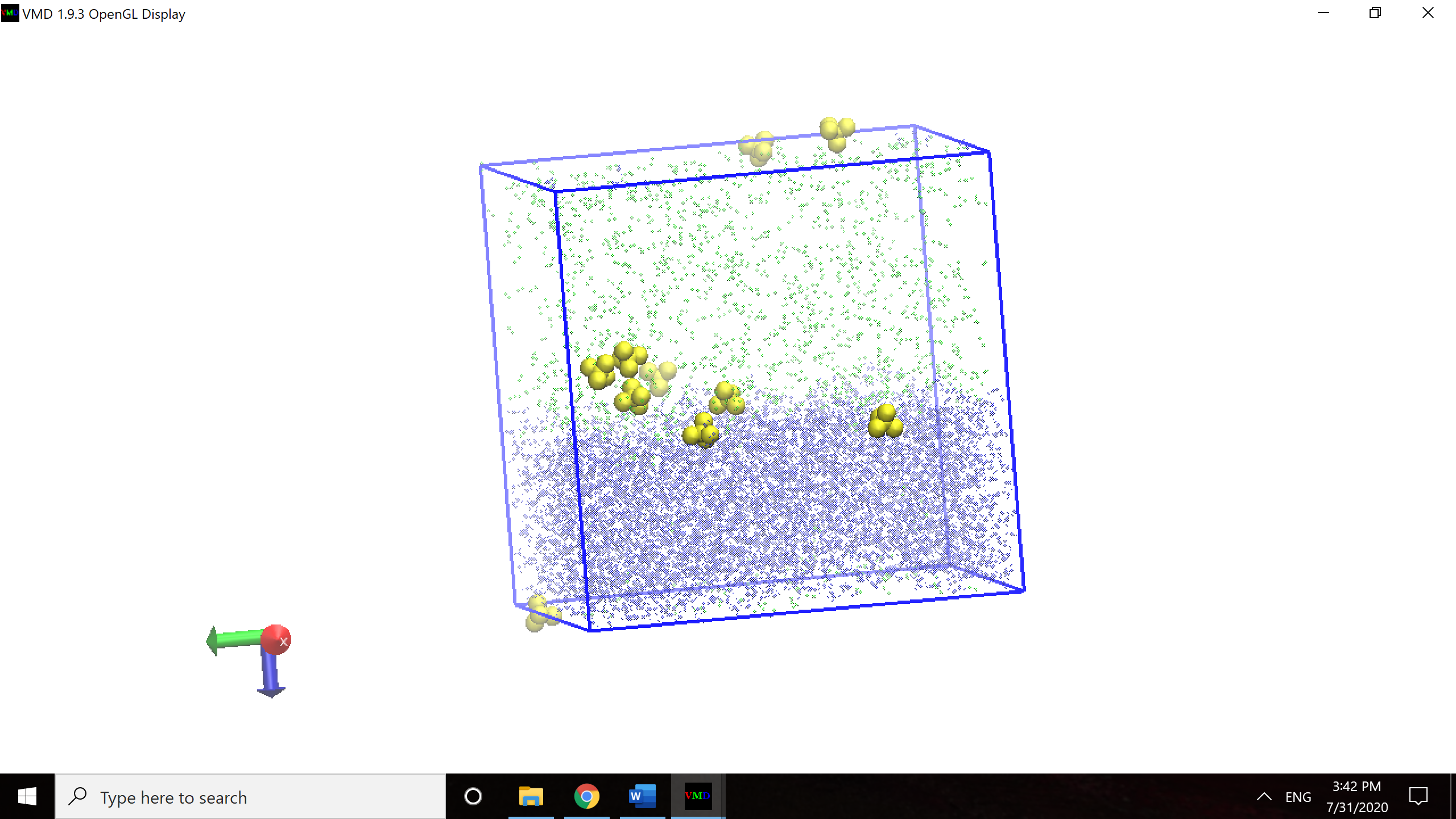  D) |

**Figure S1.4:** Illustrations of (A) System 19 with CCl_4_ (yellow) + Cr (red) with chlorine (cyan) + water (purple), (B) System 20 with CCl_4_ + Cr with chlorine + water + air (green), (C) System 21 with CCl_4_ + water, (D) System 22 with CCl_4_ + water +air after 10 ns of simulations.

| 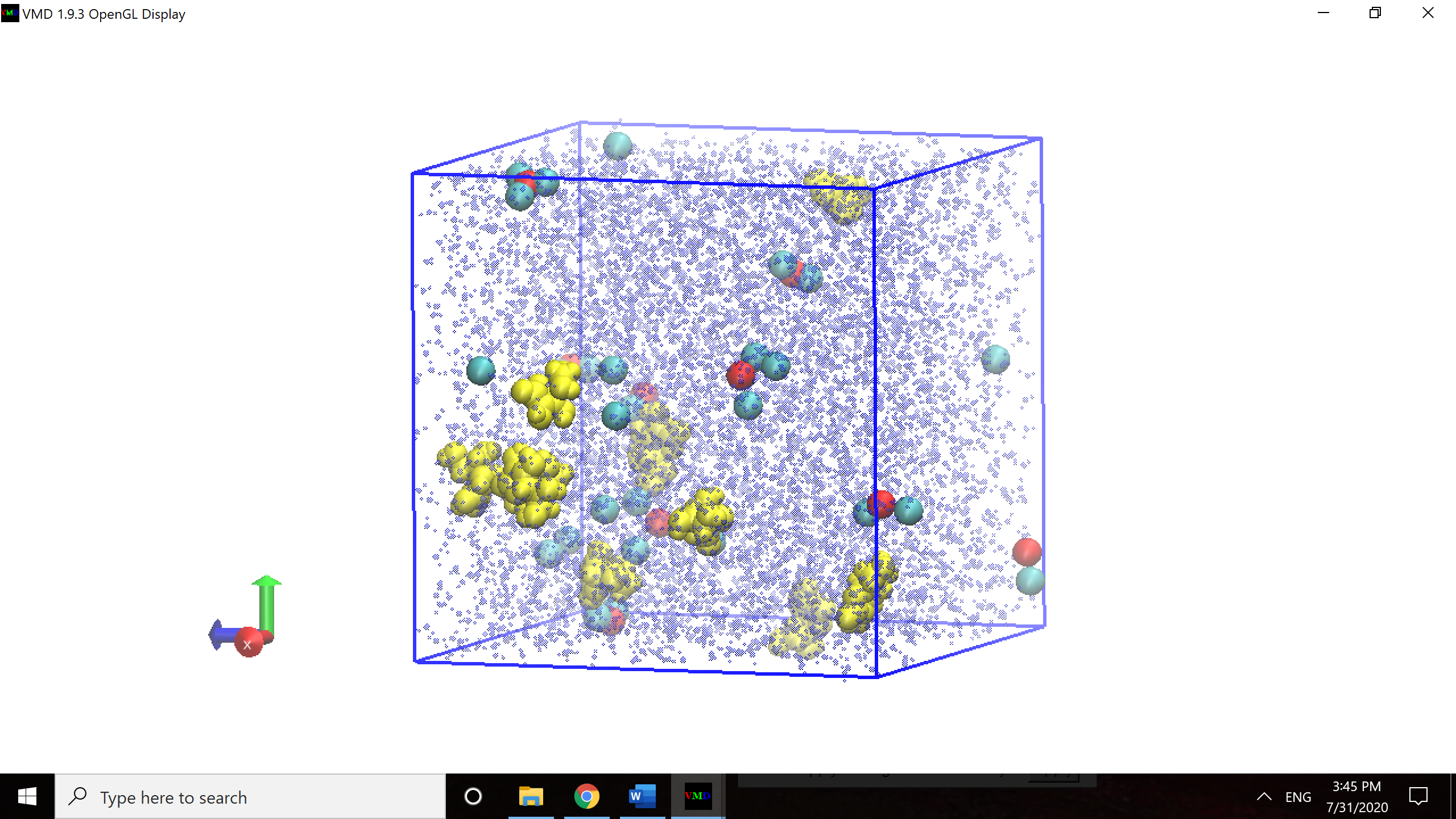  A) | 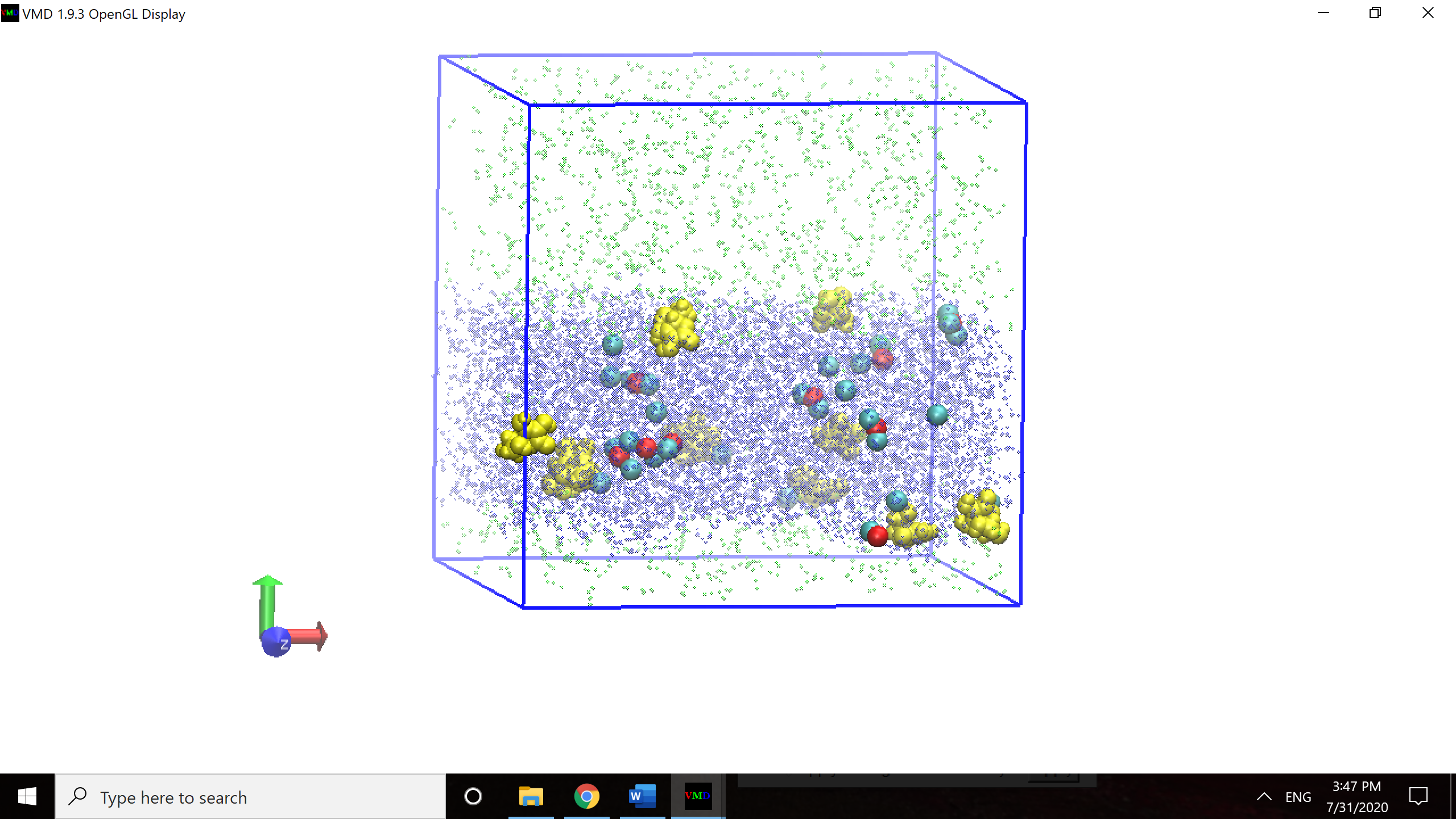  B) |
| --- | --- |
| 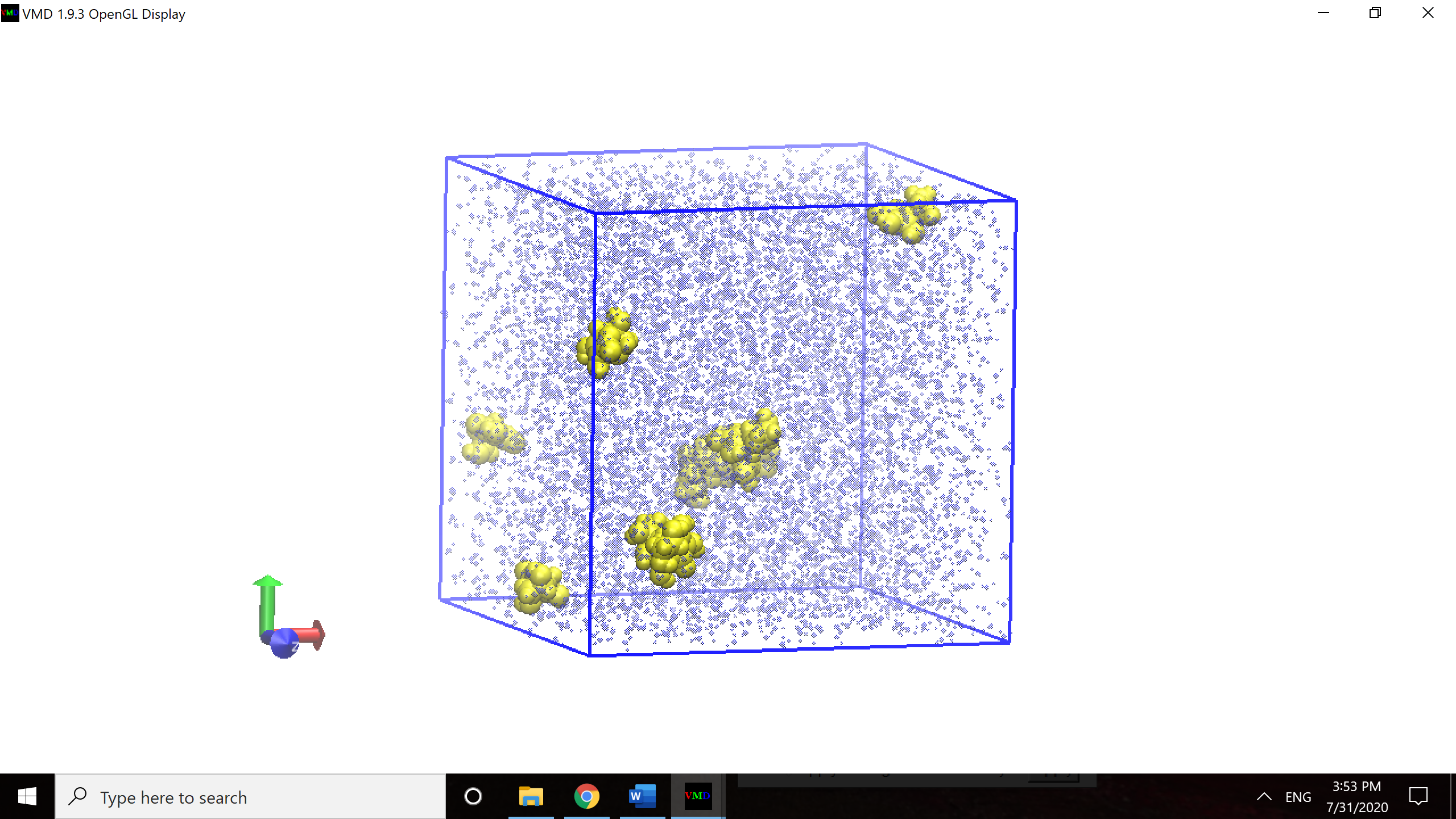  C) | 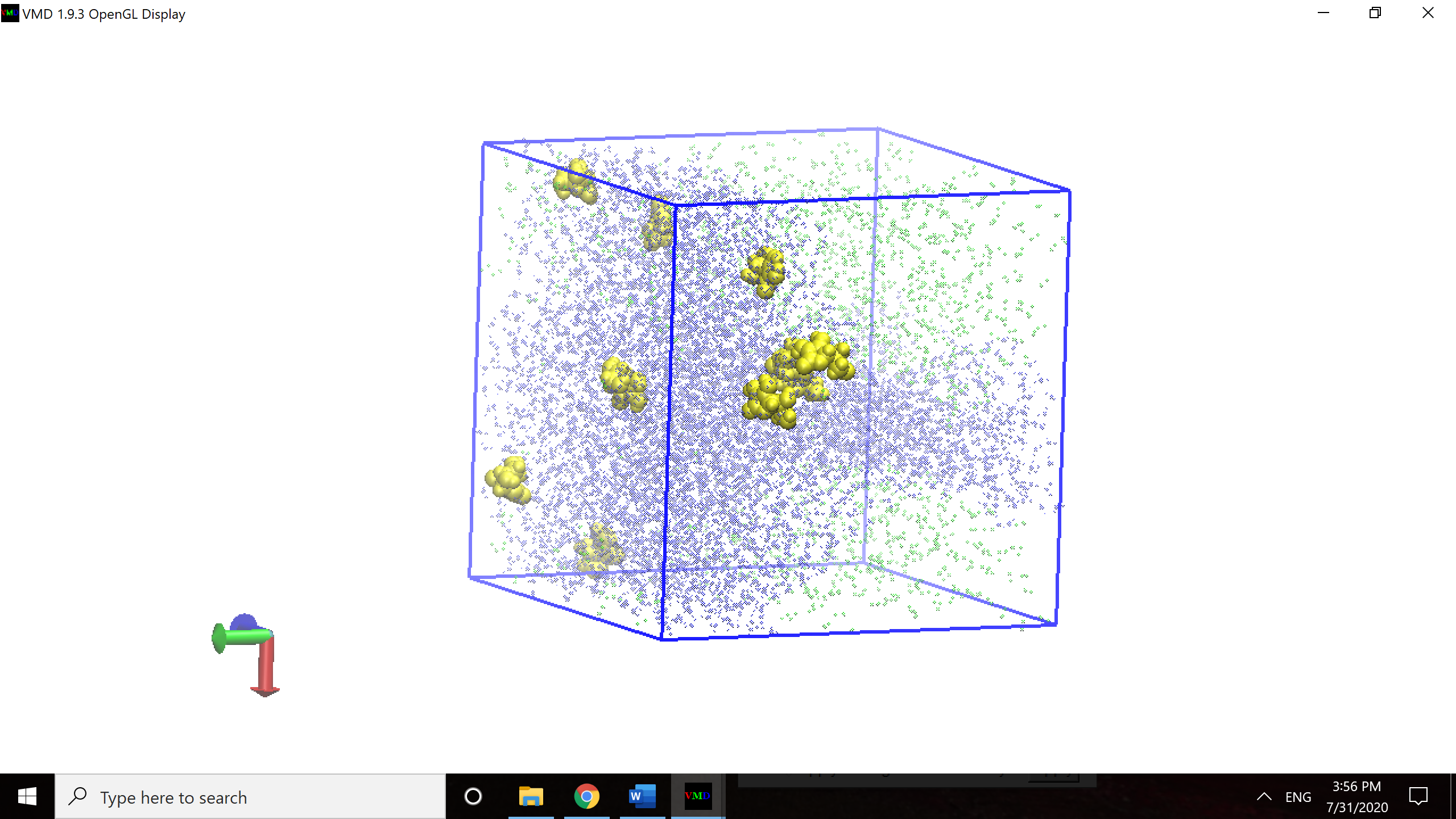  D) |

**Figure S1.5:** Illustrations of (A) System 23 with ascorbic acid (yellow) + Cr (red) with chlorine (cyan) + water (purple), (B) System 24 with ascorbic acid + Cr with chlorine + water + air (green), (C) System 25 with ascorbic acid + water, (D) System 26 with ascorbic acid + water +air after 10 ns of simulations.

| 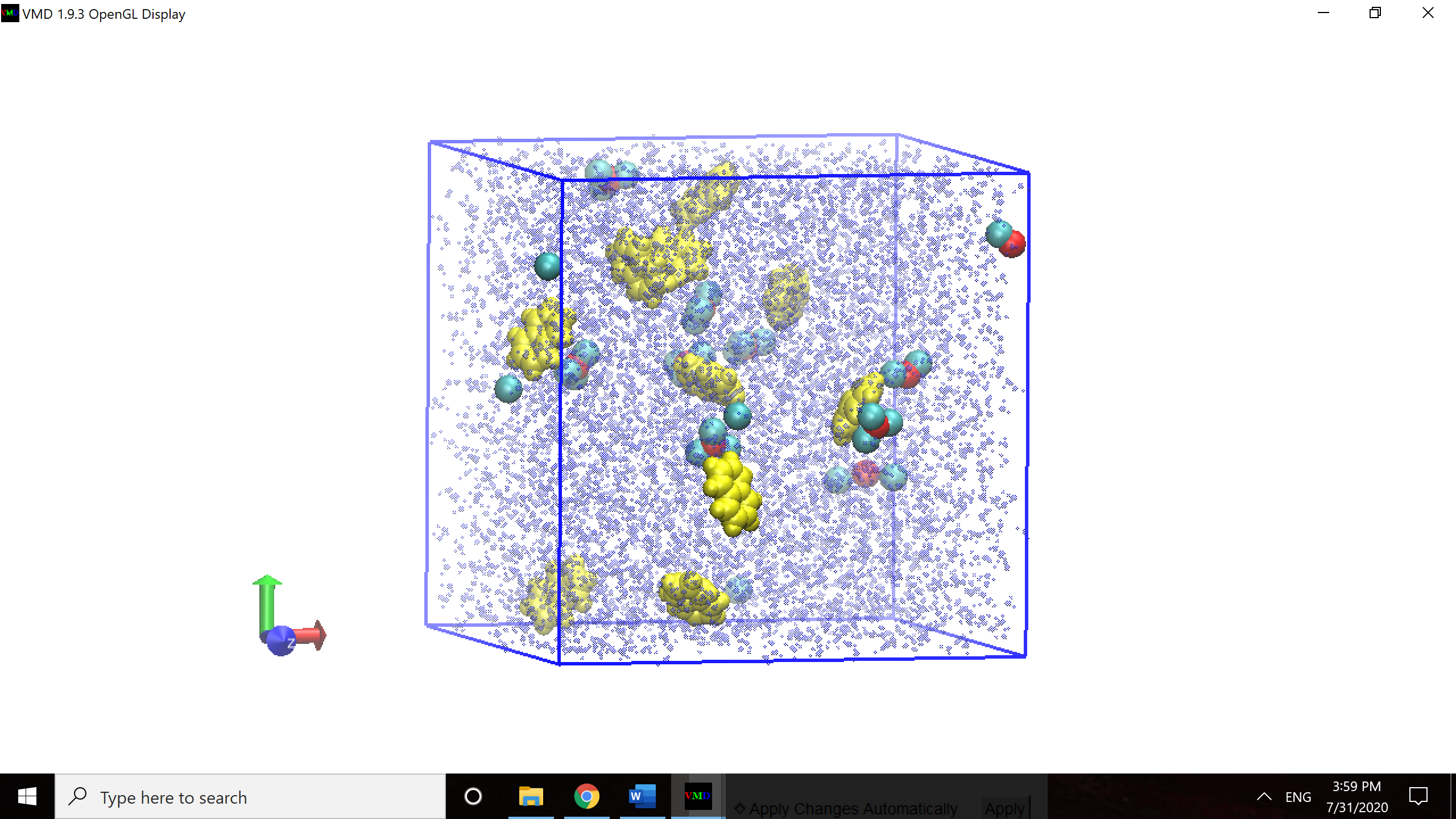  A) | 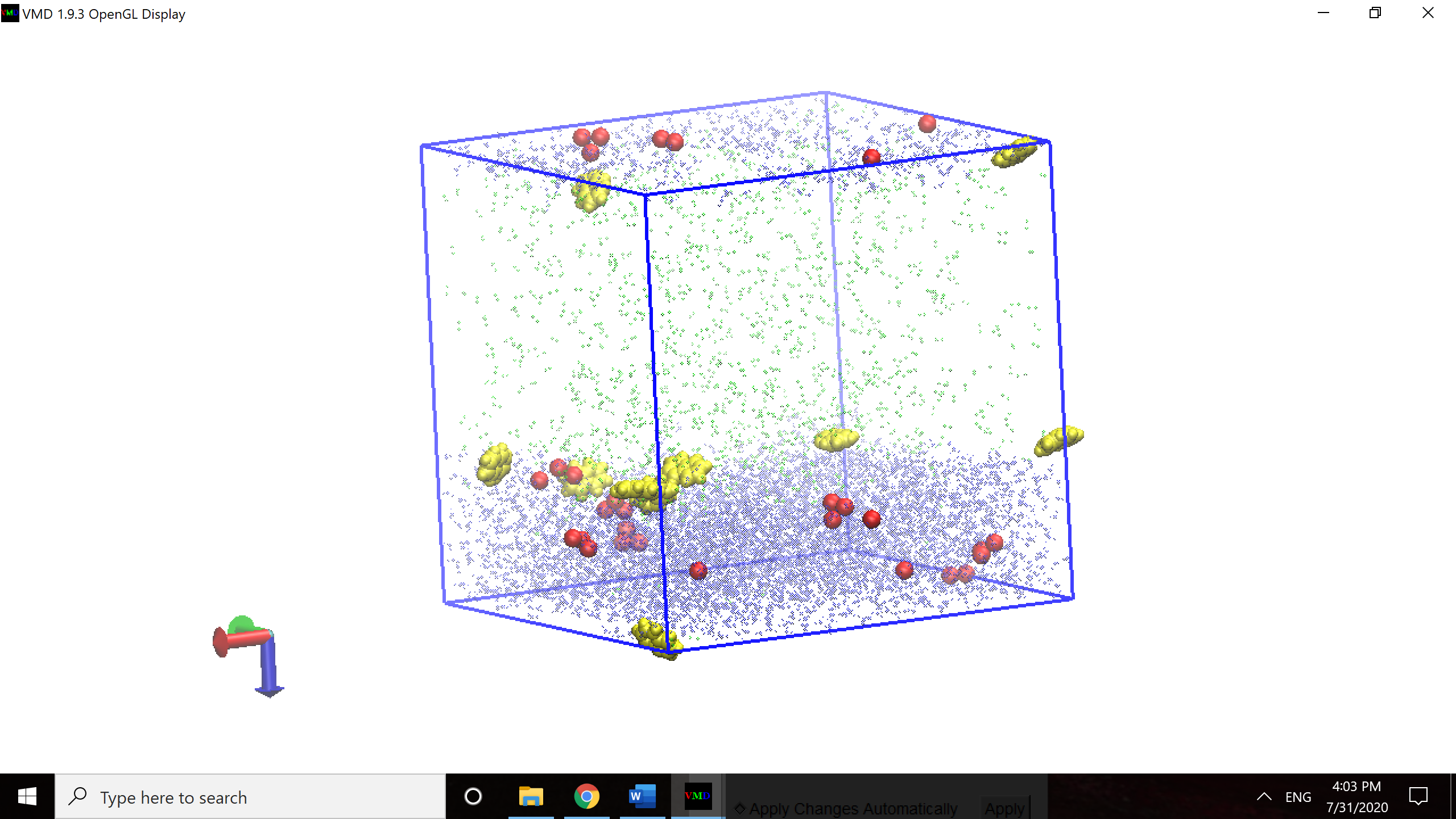  B) |
| --- | --- |
| 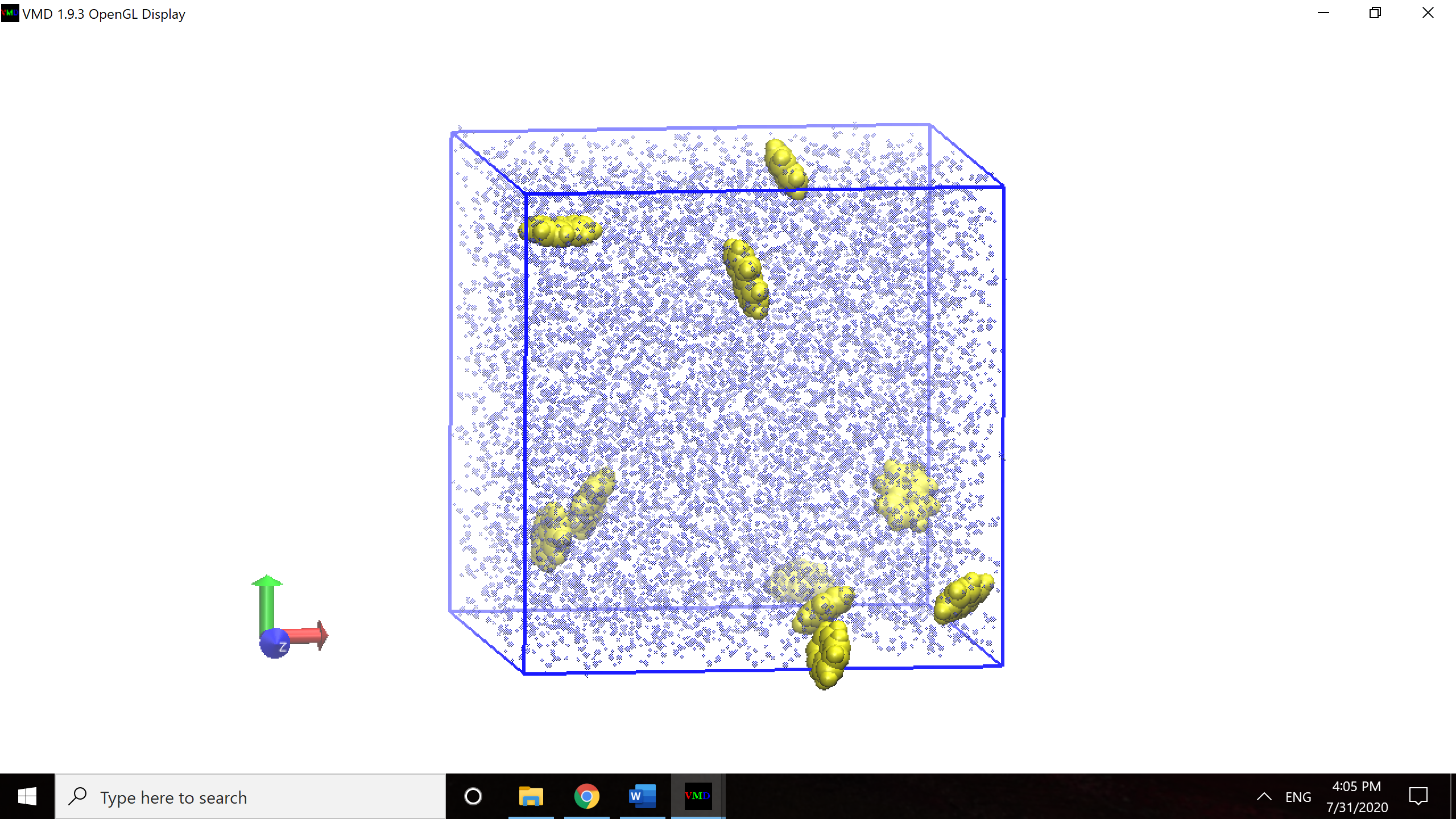  C) | 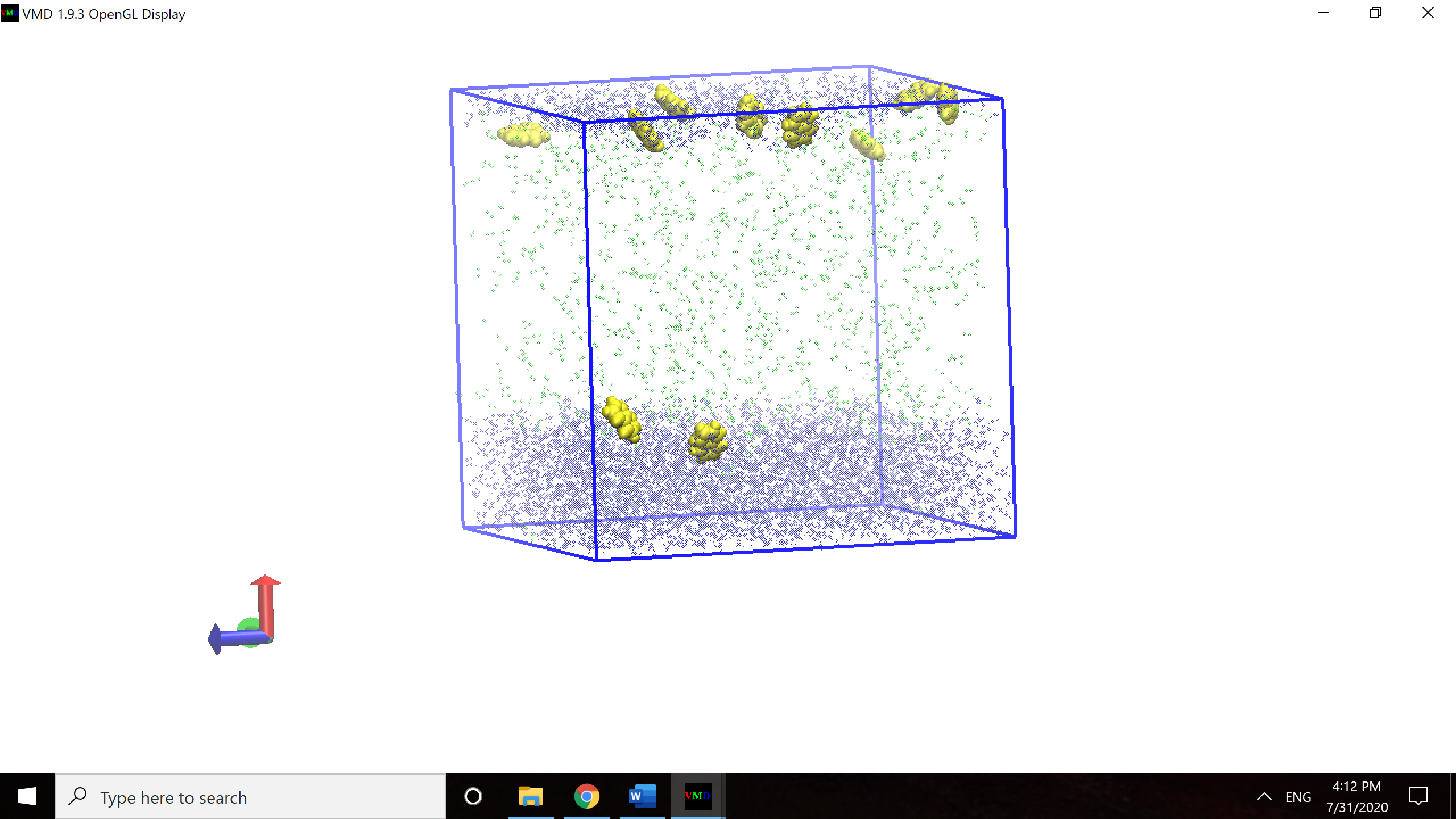  D) |

**Figure S1.6:** Illustrations of (A) System 27 with naphthalene (yellow) + Cr (red) with chlorine (cyan) + water (purple), (B) System 28 with naphthalene + Cr with chlorine + water + air (green), (C) System 29 with naphthalene + water, (D) System 30 with naphthalene + water +air after 10 ns of simulations.

| 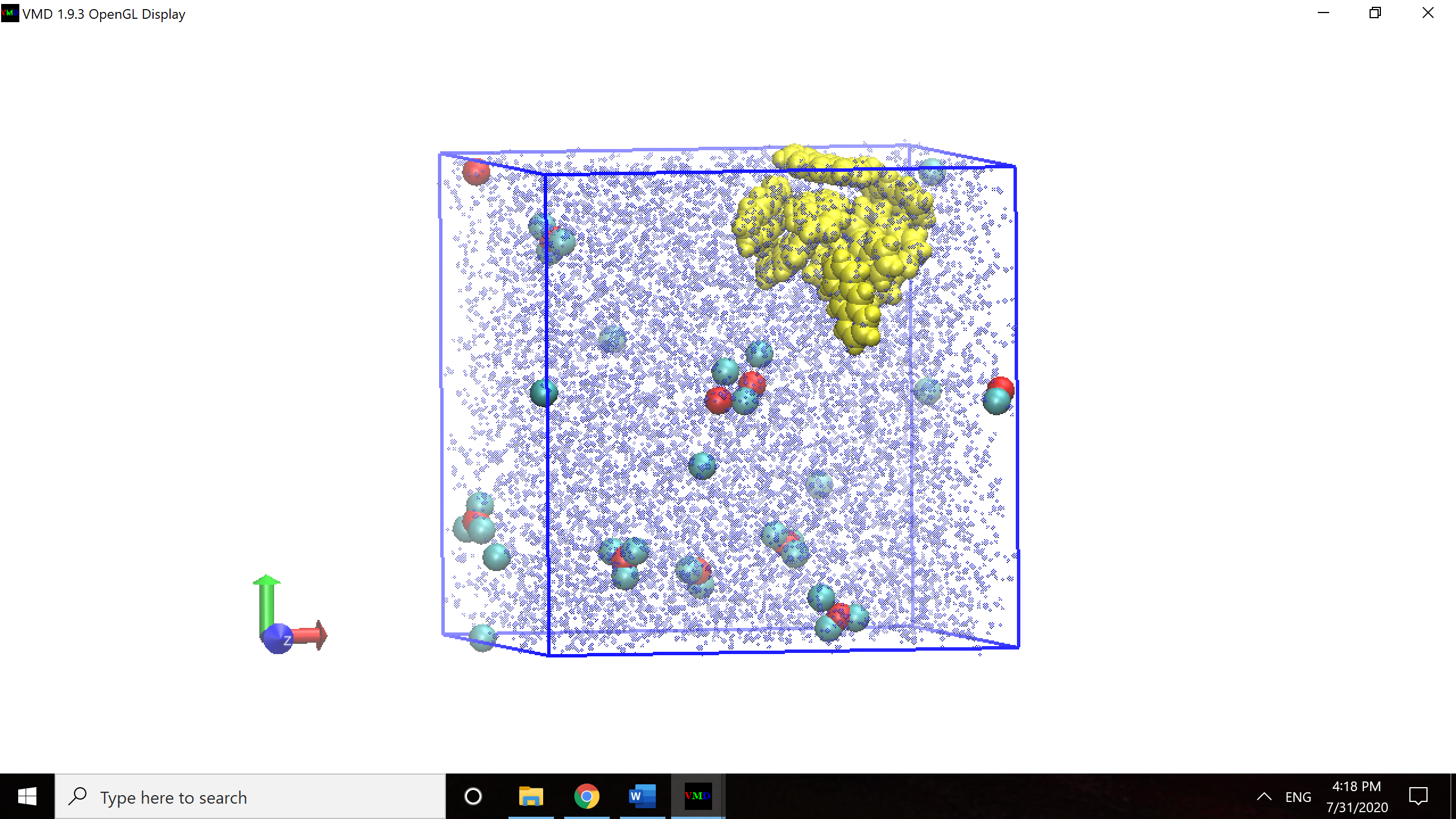  A) | 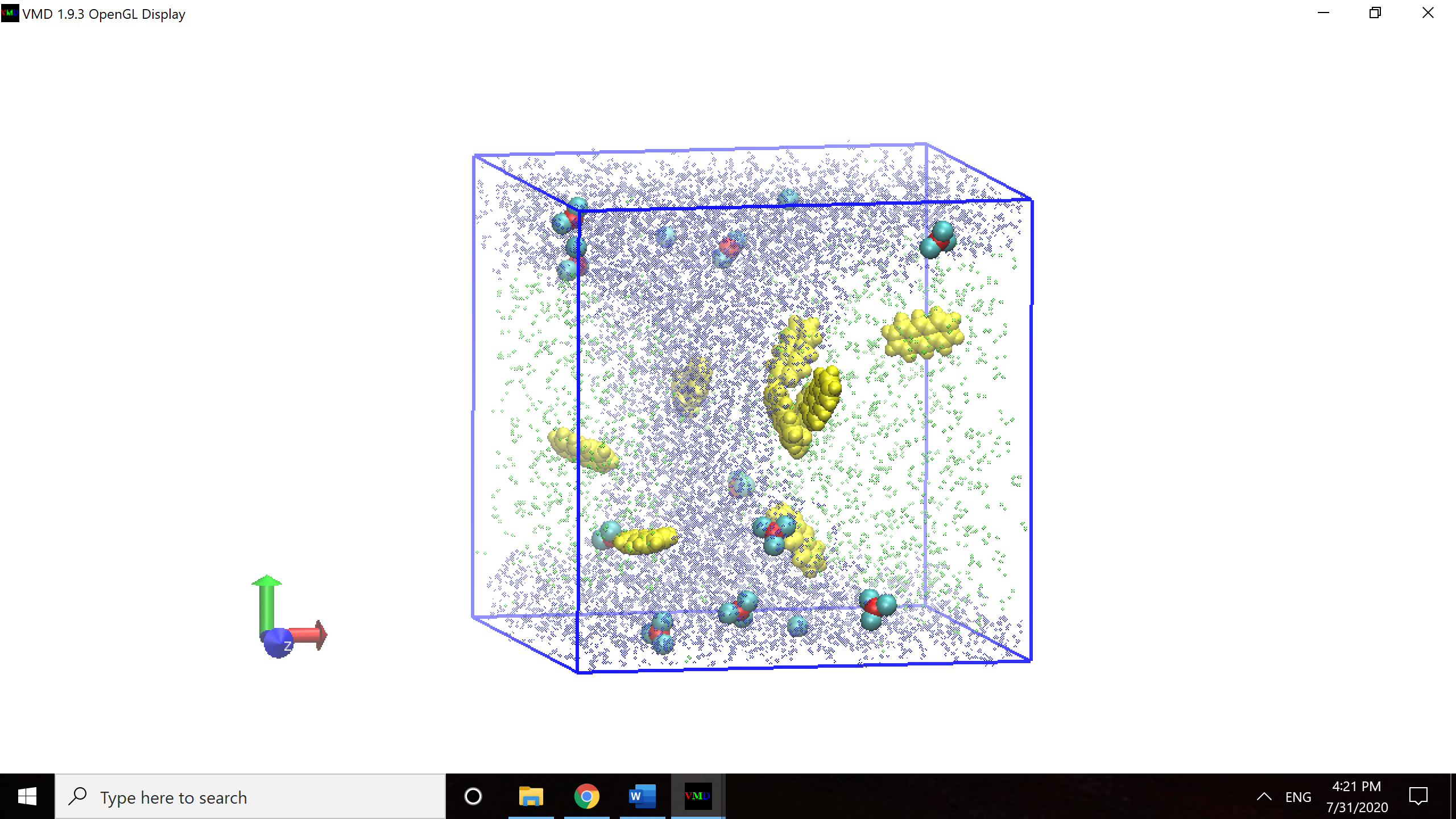  B) |
| --- | --- |
| 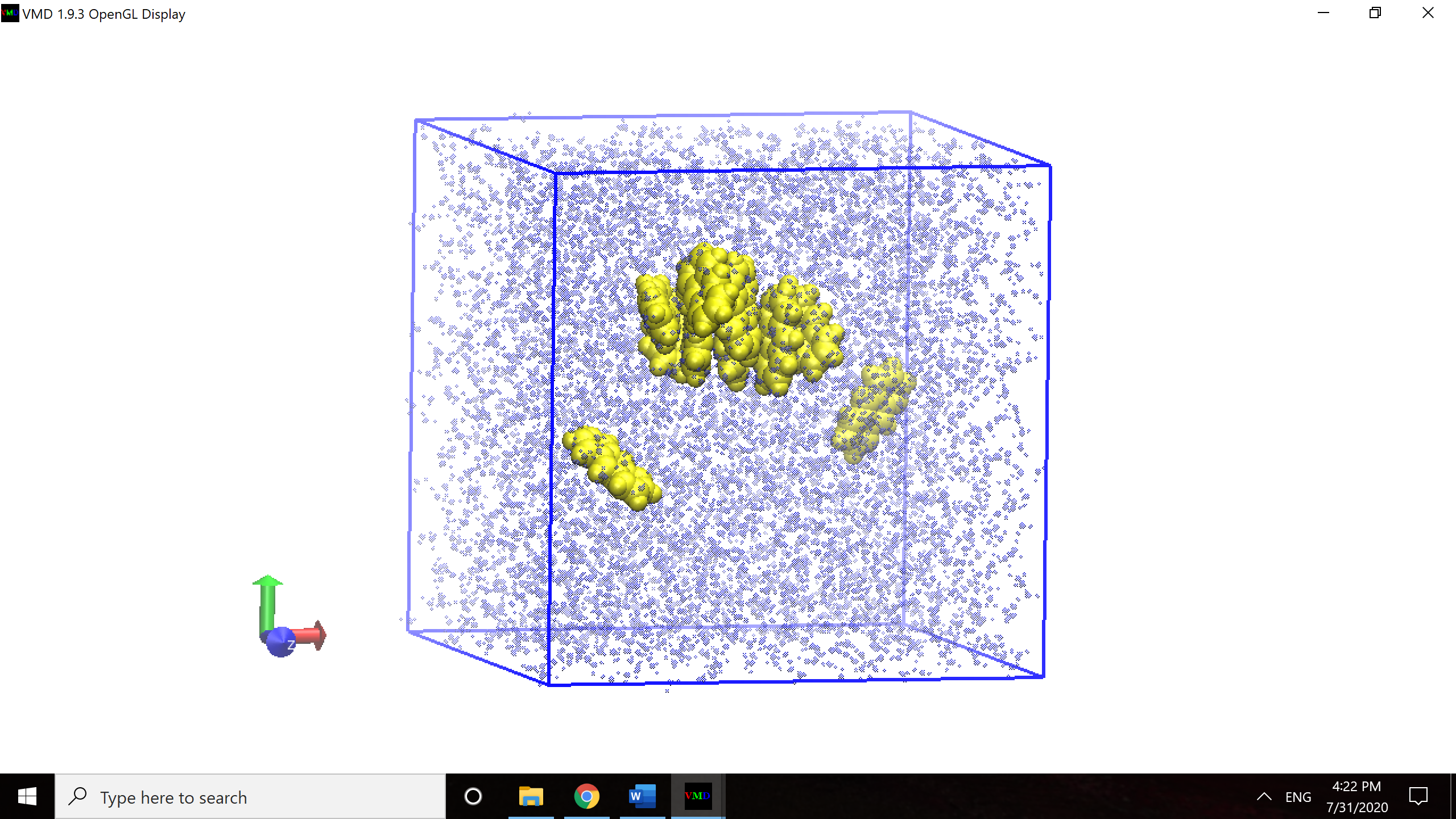  C) | 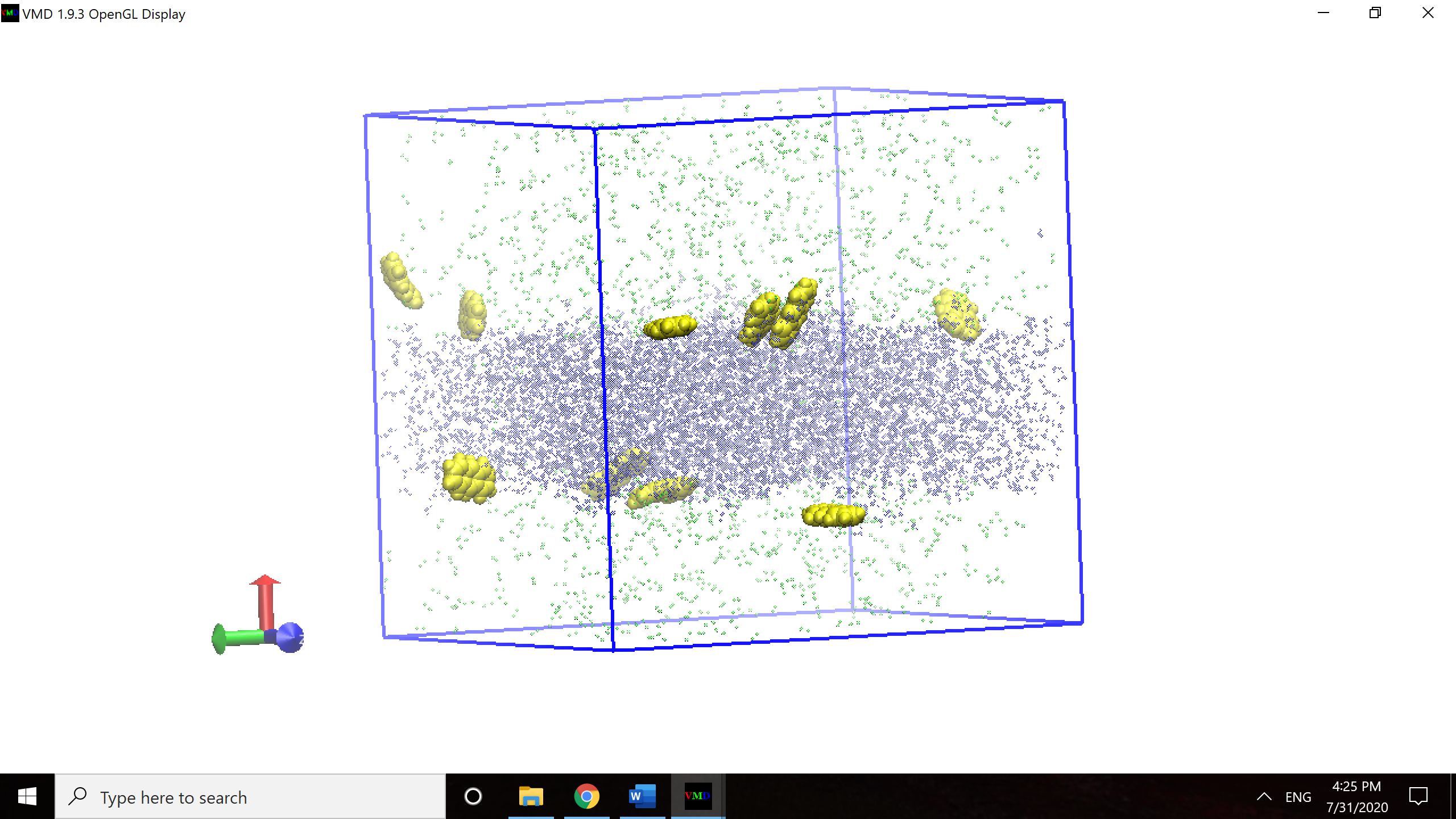  D) |

**Figure S1.7:** Illustrations of (A) System 31 with anthracene (yellow) + Cr (red) with chlorine (cyan) + water (purple), (B) System 32 with anthracene + Cr with chlorine + water + air (green), (C) System 33 with anthracene + water, (D) System 34 with anthracene + water +air after 10 ns of simulations.
